# Efficacy of fecal microbiota transplantation in patients with Parkinson’s disease: clinical trial results from a randomized, placebo-controlled design

**DOI:** 10.1080/19490976.2023.2284247

**Published:** 2023-12-06

**Authors:** Yi Cheng, Guohua Tan, Qihui Zhu, Chun Wang, Guangcong Ruan, Senhong Ying, Jinlong Qie, Xiaofei Hu, Zhifeng Xiao, Fenghua Xu, Lu Chen, Minjia Chen, Yang Pei, Hao Zhang, Yuting Tian, Dongfeng Chen, Xingyin Liu, Heqing Huang, Yanling Wei

**Affiliations:** a Department of Gastroenterology, Chongqing Key Laboratory of Digestive Malignancies, Daping Hospital, Army Medical University (Third Military Medical University), Chongqing, China; b Department of Neurology, Southwest Hospital, Army Medical University, Chongqing, China; c Department of Pathogen Biology-Microbiology Division, Key Laboratory of Pathogen of Jiangsu Province; The Affiliated Suzhou Hospital of Nanjing Medical University, Suzhou Municipal Hospital Gusu School, Nanjing Medical University, Jiangsu, China; d Department of Radiology, Southwest Hospital, Army Medical University, Chongqing, China

**Keywords:** Parkinson’s disease, clinical trial, fecal microbiota transplantation, gut microbiota, microbiota-gut-brain axis

## Abstract

The occurrence and development of Parkinson’s disease (PD) have been demonstrated to be related to gut dysbiosis, however, the impact of fecal microbiota transplantation (FMT) on microbiota engraftment in PD patients is uncertain. We performed a randomized, placebo-controlled trial at the Department of Neurology, Army Medical University Southwest Hospital in China (ChiCTR1900021405) from February 2019 to December 2019. Fifty-six participants with mild to moderate PD (Hoehn-Yahr stage 1–3) were randomly assigned to the FMT and placebo group, 27 patients in the FMT group and 27 in the placebo group completed the whole trial. During the follow-up, no severe adverse effect was observed, and patients with FMT treatment showed significant improvement in PD-related autonomic symptoms compared with the placebo group at the end of this trial (MDS-UPDRS total score, group×time effect, B = -6.56 [−12.98, −0.13], *P* < 0.05). Additionally, FMT improved gastrointestinal disorders and a marked increase in the complexity of the microecological system in patients. This study demonstrated that FMT through oral administration is clinically feasible and has the potential to improve the effectiveness of current medications in the clinical symptoms of PD patients.

## Introduction

Parkinson’s disease (PD) is a progressive neurodegenerative disease that mainly occurs in elderly individuals, with a global incidence of nearly 14 per 100,000. It is the most serious motor disorder and the second major neurodegenerative disease after Alzheimer’s disease,^1^ affecting more than 1% of the general population in people aged 60 and above and 2% of people over the age of 80.^2^ Patients with PD often suffer from a series of complicated clinical symptoms, including motor dysfunction, such as rigidity, bradykinesia, tremor, and impaired balance,^3^ and nonmotor dysfunctions, such as depression, cognitive damage, sleep disorders, and gastrointestinal malfunctions.^4^ In patients with PD, motor disorders are related to the loss of dopaminergic neurons in the substantia nigra. However, the neuropathological changes that occur in areas other than the substantia nigra area are more widespread, implicating the autonomic nervous, olfactory, and other systems.^5,6^ This pathology leads to a broad spectrum of nonmotor symptoms, which are important features of PD.

Gastrointestinal disorders, such as dysphagia and constipation, are one of the most common non-motor symptoms in PD patients.^7^ These symptoms worsen over time even if patients are being treated and gradually cause great damage to the patient’s health and daily life mobility, eventually resulting in the need for caregivers. Of particular interest, gastrointestinal symptoms and gut microbiota gained increasing attention based on the hypothesis that PD pathophysiology may be originated from the gut.^8^

The current treatment for patients with PD is symptomatic. Doctors focus on improving motor (e.g., tremor, rigidity, and bradykinesia) and nonmotor (e.g., constipation, cognition, mood, and sleep) signs and symptoms.^9^ Unfortunately, due to the complex pathogenesis of PD, the etiology is still uncertain, and no disease-modifying pharmacologic treatments are available.^10^ Currently, there are several choices for treating PD, including therapy with carbidopa-levodopa, monoamine oxidase-B inhibitors, and dopamine agonists. However, increasing evidence demonstrated that the pathology of PD doesn’t only depend on dopamine in the basal ganglia but also involves neurotransmitters in other neural regions, the endocrine system, and metabolism.^11^ The effectiveness of current medications which are mostly focused on dopaminergic neurons gradually decreases.

Recent studies have revealed that the bidirectional communication system between the brain and gut, known as the gut-brain axis, provides insight into neurological disorders’ etiology and physiology.^12^ Increasing evidence in clinical and animal studies has shown that gut microbiota is an important regulator of brain development and human behavior.^4^ In recent years, more and more reports have shown that the gut microbiota composition of patients with PD is different from that of healthy people, suggesting a state of microbiota imbalance.^13,14^ Particularly, butyrate-producing bacteria related to anti-inflammatory pathways, such as *Blautia*, *Coprococcus*, and *Roseburia*, have a relatively lower abundance in the stool of patients with PD, while significant alteration in metabolic pathways as well as gut microbiota products were found in PD samples.^15^ Several risks (physical activity, constipation, subthreshold parkinsonism, and physical activity) and prodromal markers are also associated with alterations in gut microbiota composition.^16,17^

In the past decade, it has been identified that gut microbiota can regulate the pathophysiology of synucleinopathies in PD murine model,^18^ and probiotics have a remarkable effect on relieving PD symptoms, especially constipation.^19^ Sun et al. reported that fecal microbiota transplantation (FMT) from healthy mice could significantly improve motor function and striatal neurotransmitters and decrease neuroinflammation in PD murine models through the TLR4/TNF-α signaling pathway.^20^ In addition, a preliminary clinical study and serial case reports also showed that FMT via colonoscopy and nasal-jejunal tube-induced symptom improvement in PD participants with minimal and self-recovering adverse effects.^21–23^ However, these reports involved a small number of participants and did not include those who received a placebo. Hence, further clinical research is needed to determine whether FMT is effective in improving the clinical symptoms of PD.

In the present study, to reveal the efficacy and safety of FMT in improving the clinical symptoms of PD patients through oral administration (FMT capsule patent NO.: ZL201510304041.4), we performed a placebo-controlled randomized clinical trial for 54 patients with PD associated with gastrointestinal disorders. Our work provides new evidence for using FMT as an auxiliary intervention for the management of PD.

## Results

### Information of patients

**Figure 1.**
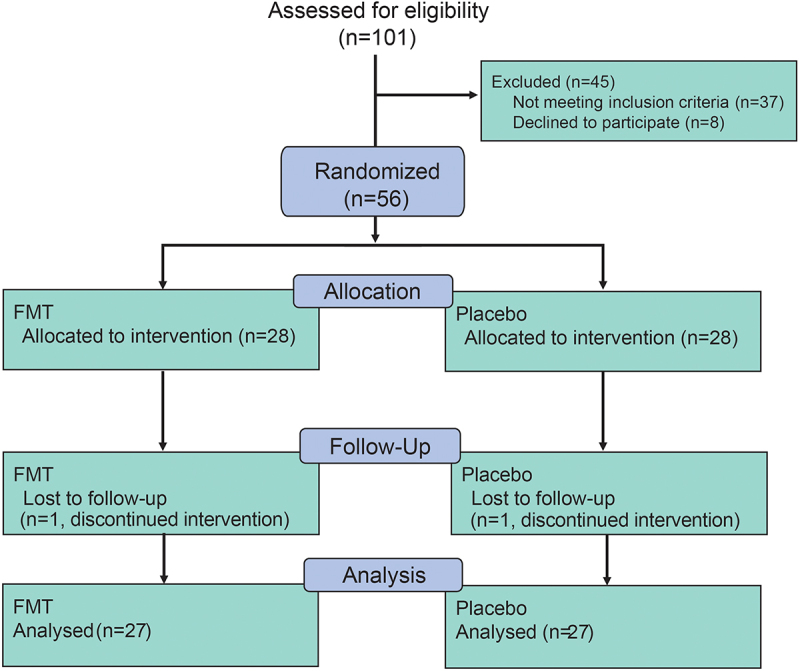
Flow chart of patients included and excluded in this trial.

As shown in Figure 1, 101 patients were screened, and 56 patients were enrolled in this trial at the Department of Neurology, Army Medical University Southwest Hospital, from February 2019 to December 2019. In total, 54 patients (27 in the FMT group and 27 in the placebo group) finished the whole trial, while one patient from the FMT arm and one from the placebo arm were lost to follow-up (Figure 1). Finally, data from 54 patients who completed the whole trial were used for statistical and bioinformatics analysis. Baseline information of demographics and clinical manifestations were presented in Table 1. Demographic characteristics were similar between the two arms, except for the history of drinking and smoking. The average age of enrolled participants was 61.57, and more than half of them were male (about 59.26% male). Participants in the placebo group used to have a higher rate of smoking history and alcohol drinking history, while constipation and laxative usage were similar in both groups.

**Table 1.** · baseline characteristics of participants.

Characteristics	FMT	Placebo
n	27	27
**Demographics**		
Age, mean (SD)	60·52 (8·68)	62·63 (8·41)
Male sex, n (%)	15 (55·56)	17 (62·96)
BMI, mean (SD)	22·33 (2·16)	22·43 (2·16)
History of smoking, %	7·41	18·52
History of drinking alcohol, %	3·70	14·81
Taking laxative, %	37·04	37·04
Constipation, %	85·19	88·89
Years since PD diagnosis, mean (SD)	6·74 (4·00)	5·85 (4·35)
**Baseline assessment scores**		
Bristol score, median (IQR)	2 (1,4)	2 (1,3)
Stool frequency (time/week), median (IQR)	1 (1,2)	2 (1,2)
IBS-SSS score, mean (SD)	133·37 (72·35)	128·52 (60·36)
IBS-QOL score, mean (SD)	21·96 (14·81)	18·67 (11·80)
GSRS score, mean (SD)	7·89 (2·89)	7·30 (3·14)
PD-QOL score, mean (SD)	195·33 (34·23)	200·96 (46·46)
PHQ-9 score, mean (SD)	7·30 (4·28)	7·74 (4·93)
GAD-7 score, mean (SD)	3·74 (3·22)	4·44 (4·25)
GDS-15 score, mean (SD)	5·59 (2·65)	6·26 (4·70)
MOCA score, mean (SD)	19·41 (5·23)	19·30 (5·55)
MMSE score, mean (SD)	25·44 (4·21)	26·15 (3·44)
Total score MDS-UPDRS, mean (SD)	51·33 (16·92)	60·15 (17·74)
MDS-UPDRS part 1 score, mean (SD)	11·11 (4·42)	12·11 (4·89)
MDS-UPDRS part 2 score, mean (SD)	12·04 (4·78)	14·74 (4·89)
MDS-UPDRS part 3 score, mean (SD)	24·74 (10·58)	29·59 (13·07)
MDS-UPDRS part 4 score, mean (SD)	3·44 (3·26)	3·70 (3·20)
**Concomitant therapy at baseline**		
Oral levodopa-based therapy, n (%)	19 (70·37)	20 (74·07)
Dopamine agonist, n (%)	17 (62·96)	14 (51·85)
MAO-B inhibitor, n (%)	4 (14·81)	0 (0·00)
COMT inhibitor, n (%)	6 (22·22)	2 (7·41)
Amantadine, n (%)	1 (3·70)	3 (11·11)
Anticholinergic, n (%)	1 (3·70)	3 (11·11)
Antidepressant/Anti-anxiety, n (%)	0 (0·00)	0 (0·00)
Hypnotics, n (%)	0 (0·00)	0 (0·00)
Incontinence, n (%)	0 (0·00)	1 (3·70)
Blood Pressure, n (%)	0 (0·00)	0 (0·00)
Deep Brain Stimulation, n (%)	0 (0·00)	0 (0·00)

After the treatment schedule is completed, participants were followed up on 4th, 8th, and 12th week. During follow-up, only a small number of patients showed mild adverse events (AEs) after the intervention, and no severe adverse event occurred (Table 2).

**Table 2.** Adverse effects occurred in participants.

Adverse effects	Overall	FMT	Placebo
All events (n)	6	3	3
Stomach ache (n)	0	0	0
Nausea (n)	1	1	0
Headache (n)	0	0	0
Fatigue (n)	0	0	0
Dizziness (n)	0	0	0
Bloating (n)	1	0	1
Flatulence (n)	3	1	2
Diarrhea (n)	1	1	0
Mucus in the stool (n)	0	0	0
Obstipation (n)	0	0	0
Reflux (n)	0	0	0
Rash (n)	0	0	0
Vomiting (n)	0	0	0
Fever sensation (n)	0	0	0
Chest pain (n)	0	0	0
Fever (n)	0	0	0
Influenza symptoms (n)	0	0	0
Uncomfortable (n)	0	0	0

### FMT significantly improved the PD-associated symptoms in patients

PD is associated with a high diversity of motor and nonmotor symptoms, and the evaluation of four dimensions by MDS-UPDRS scales, including total score and parts 1, 2, 3, and 4 performed at baseline and the 4th, 8th, and 12th weeks after the intervention, as the primary outcome for monitoring the progression of PD. As Table 3 illustrated, participants in two arms have similar MDS-UPDRS total scores at baseline (group effect, B = −8.81 [−17.89, 0.26], *P* = 0.06), but a significantly larger decrease of the MDS-UPDRS total score was observed in the FMT arm at week 12 (group×time effect, B = −6.56 [−12.98, −0.13], *P* < 0.05), implicating that combination of FMT and conventional PD treatments induced a better improvement in clinic symptoms.

**Table 3.** Clinic outcomes of participants of FMT and placebo arms.

		Score		Group effect^d^		Time effect^e^		Group×time effect^f^	
Outcomes		FMT arm	Placebo arm	B (95% CI)	P	B (95% CI)	P	B (95% CI)	P
MDS-UPDRS^a^	Week0	51.33 (16.92)	60.15 (17.74)	−8·81 (−17.89, 0.26)	0·06	NA	NA	NA	NA
	Week4	43.33 (16.49)	55.52 (19.16)			−4·63 (−7.89, −1.38)	**	−3·37 (−8.60, 1.86)	0·21
	Week8	42.85 (15.93)	53.26 (17.84)			−6·89 (−12.15, −1.63)	*	−1·59 (−8.28, 5.10)	0·64
	Week12	41.93 (19.44)	57.3 (15.27)			−2·85 (−7.03, 1.33)	0·18	−6·56 (−12.98, −0.13)	*
MDS-UPDRS 1^a^	Week0	11.11 (4.42)	12.11 (4.89)	−1·00 (−3.44, 1.44)	0·42	NA	NA	NA	NA
	Week4	8.33 (3.27)	11.63 (5.06)			−0·48 (−1.69, 0.73)	0·44	−2·30 (−4.22, −0.37)	*
	Week8	8.52 (4.41)	11.3 (5.27)			−0·81 (−2.46, 0.83)	0·33	−1·78 (−4.09, −0.37)	0·13
	Week12	8.37 (5.06)	11.56 (4.85)			−0·56 (−1.76, 0.65)	0·37	−2·19 (−4.27, −0.10)	*
MDS-UPDRS 2^a^	Week0	12.04 (4.78)	14.74 (4.03)	−2·70 (−5.02, −0.39)	*	NA	NA	NA	NA
	Week4	11.04 (5.06)	14.52 (5.18)			−0·22 (−1.53, 1.08)	0·74	−0·78 (−2.60, 1.04)	0·40
	Week8	10.67 (5.26)	13.74 (5.45)			−1·00 (−2.52, 0.52)	0·20	−0·37 (−2.24, 1.50)	0·70
	Week12	10.04 (5.52)	14.3 (4.99)			−0·44 (−1.61, 0.72)	0·45	−1·56 (−3.16, 0.05)	0·06
MDS-UPDRS 3^a^	Week0	24.74 (10.58)	29.59 (13.07)	−4·85 (−11.08, 1.37)	0·13	NA	NA	NA	NA
	Week4	21.37 (12.84)	25.74 (12.75)			−3·85 (−5.58, −1.85)	***	0·48 (−3.47, 4.43)	0·81
	Week8	20.67 (9.63)	24.44 (11.15)			−5·15 (−8.52, −1.78)	**	1·07 (−3.33, 5.48)	0·63
	Week12	20.89 (11.86)	27.48 (9.54)			−2·11 (−5.22, 1.00)	0·18	−1·74 (−6.06, 2.58)	0·43
MDS-UPDRS 4^a^	Week0	3.44 (3.26)	3.7 (3.2)	−0·26 (−1.95, 1.43)	0·76	NA	NA	NA	NA
	Week4	2.59 (2.37)	3.63 (3.52)			−0·07 (−0.84, 0.69)	0·85	−0·78 (−1.92, 0.37)	0·18
	Week8	3.00 (2.34)	3.78(3.83)			0·07 (−1.00, 1.15)	0·89	−0·52 (−1.90, 0.86)	0·46
	Week12	2.63 (2.34)	3.96 (3.01)			0·26 (−0.51, 1.03)	0·51	−1·07 (−2.33, 0.18)	0·09
IBS-SSS^a^	Week0	133.37 (72.35)	128.52 (60.36)	4·85 (−30.03, 39.73)	0·79	NA	NA	NA	NA
	Week4	55.19 (65.83)	91.85 (72.38)			−36·67 (−61.00, −12.33)	**	−41·52 (−72.08, −10.96)	**
	Week8	72.15 (72.04)	89.63 (69.92)			−38·89 (−58.48, −19.29)	***	−22·33 (−54.35, 9.68)	0·17
	Week12	64.74 (59.77)	100.74 (64.15)			−27·78 (−43.76, −11.80)	**	−40·85 (−71.58, −10.13)	**
GSRS^a^	Week0	7.89 (2.89)	7.3 (3.14)	0·59 (−0.99, 2.17)	0·46	NA	NA	NA	NA
	Week4	4.02 (3.44)	6.3 (3.9)			−1·00 (−1.82, −0.19)	*	−2·87 (−4.10, −1.64)	***
	Week8	3.85 (2.9)	6.48 (4.15)			−0·81 (−1.92, −0.29)	0·15	−3·22 (−4.77, −1.68)	***
	Week12	3.89 (3.11)	6.33 (3.33)			−0·96 (−1.76, −0.17)	*	−3·04 (−4.32, −1.76)	***
IBS-QOL^a^	Week0	21.96 (14.81)	18.67 (11.8)	3·30 (−3.71, 10.31)	0·36	NA	NA	NA	NA
	Week4	8.7 (8.8)	16.89 (17.21)			−1·78 (−5.81, 2.25)	0·39	−11·48 (−17.52, −5.45)	***
	Week8	8.26 (7.7)	17.48 (14.53)			−1·19 ()-4.99, 2.62P	0·54	−12·52 (−18.85, −6.19)	***
	Week12	8.48 (8.9)	19.56 (17.39)			0·89 (−3.11, 4.89)	0·66	−14·37 (−20.65, −8.09)	***
Bristol score^b,c^	Week0	2 (1,4)	2 (1,3)	−0·15 (−1.24, 0.93)	0·08	NA	NA	NA	NA
	Week4	4 (3,4)	3 (2,4)			1·21 (0.37, 2.04)	**	0·99 (−0.21, 2.19)	0·10
	Week8	4 (3,4)	3 (2,4)			1·27 (0.41, 2.13)	**	0·81 (−0.34, 1.97)	0·17
	Week12	4 (3,4)	3 (2,4)			1·07 (0.36, 1.78)	**	0·58 (−0.36, 1.51)	0·23
Stool frequency (times per week)^b^	Week0	1 (1,2)	2 (1,2)	−0·38 (−1.40, 0.64)	0·47	NA	NA	NA	NA
	Week4	4 (3,7)	3 (2,4)			0·93 (0.33, 1.53)	**	1·37 (0.51, 2.22)	**
	Week8	4 (3,7)	2 (1,6)			0·73 (0.23, 1.24)	**	1·60 (0.72, 2.49)	***
	Week12	4 (2,7)	2 (1,4)			0·54 (0.12, 0.97)	*	1·69 (0.84, 2.54)	***
MMSE^a^	Week0	25.44 (4.21)	26.15 (3.44)	−0·70 (−2.72, 1.31)	0·49	NA	NA	NA	NA
	Week4	27.33 (3.22)	26.26 (3.56)			0·11 (−0.88, 1.10)	0·83	1·78 (0.45, 3.10)	**
	Week8	26.81 (3.76)	26.41 (3.31)			0·26 (−0.56, 1.08)	0·54	1·11 (−0.23, 2.46)	0·11
	Week12	27.22 (3.67)	26.93 (3.16)			0·78 (−0.03, 1.59)	0·06	1·00 (−0.29, 2.29)	0·13
MOCA^a^	Week0	19.41 (5.23)	19.3 (5.55)	0·11 (−2.71, 2.93)	0·94	NA	NA	NA	NA
	Week4	21.22 (4.85)	21.11 (4.5)			1·82 (0.41, 3.22)	*	0·00 (−1.97, 1.97)	1·00
	Week8	22.74 (4.83)	21.22 (4.8)			1·93 (0.70, 3.15)	**	1·41 (−0.31, 3.12)	0·11
	Week12	23.3 (4.37)	20.93 (5.2)			1·63 (0.55, 2.71)	**	2·26 (0.63, 3.89)	**
PHQ-9^a^	Week0	7.3 (4.28)	7.74 (4.93)	−0·44 (−2.86, 1.97)	0·72	NA	NA	NA	NA
	Week4	6.41 (3.68)	7.59 (4.99)			−0·15 (−1.59, 1.3)	0·84	−0·74 (−2.76, 1.28)	0·47
	Week8	7.22 (5.14)	7.52 (4.85)			−0·22 (−1.62, 1.18)	0·76	0·15 (−2.22, 2.51)	0·90
	Week12	6.37 (5.38)	7.56 (4.78)			−0·19 (−1.26, 0.89)	0·74	−0·74 (−2.79, 1.31)	0·48
GDS-15^a^	Week0	5.59 (2.65)	6.26 (4.7)	−0·67 (−2.66, 1.33)	0·51	NA	NA	NA	NA
	Week4	4.96 (3.29)	6.70 (4.35)			0·44 (−0.75, 1.63)	0·46	−1·07 (−2.63, 0.48)	0·18
	Week8	4.56 (3.21)	6.00 (4.31)			−0·26 (−1.66, 1.14)	0·72	−0·78 (−2.54, 0.98)	0·39
	Week12	4.3 (2.98)	6.04 (4.31)			−0·22 (−1.33, 0.89)	0·70	−1·07 (−2.65, 0.50)	0·18
GAD-7^a^	Week0	3.74 (3.22)	4.44 (4.25)	−0·70 (−2.68, 1.27)	0·48	NA	NA	NA	NA
	Week4	3.15 (3.24)	4.00 (4.02)			−0·44 (−1.87, 0.98)	0·54	−0·15 (−2.12, 1.83)	0·88
	Week8	3.63 (3.93)	4.33 (4.06)			−0·11 (−1.31, 1.09)	0·86	0·00 (−1.95, 1.95)	1·00
	Week12	3.00 (3.75)	4.78 (4.59)			0·33 (−1.38, 2.04)	0·70	−1·07 (−3.37, 1.22)	0·36

^a^Data were presented as mean (SD), linear model was used in GEE analysis.

^b^Data were presented as median (IQR), ordinal logistic model was used in GEE analysis.

^c^Bristol score stands for the stool characteristics.

^d^Group effect represents the difference at baseline between two arms.

^e^Time effect represents change of measurement in the placebo arm compared with baseline.

^f^Group × time effect represents additional change of measurement in the FMT arm compared with placebo arm (additional change: the difference of measurement changes compared to baseline in two arms).

*P <.05.

**P <.01.

***P <.001.

During the follow-up, the placebo arm showed a significant decrease in MDS-UPDRS total score at week 4 (time effect, B = −4.63 [−7.89, −1.38], *P* <.01) and week 8 (time effect, B = −6.89 [−12.15, −1.63] *P* <.05), compared to baseline respectively, but the MDS-UPDRS total score of the placebo arm at week 12 was similar with baseline (time effect, B = −6.56 [−12.98, −0.13], *P* =.18). As for the FMT arm, the change of MDS-UPDRS total score (compared to baseline) is similar to the placebo arm at week 4 (group×time effect, B = −3.37 [−8.60, 1.86], *P* =.21) and week 8 (group×time effect, B = −1.59 [−8.28, 5.10], *P* =.64), but decreased significantly more than the placebo arm at week 12. This result implicated that at weeks 4 and 8, both arms showed significant improvement according to the change in the MDS-UPDRS total score, but the effect of placebo intervention in participants might gradually wear off at week 12, while the effect of FMT intervention was kept till the end of this trial.

The part 1 scores of MDS-UPDRS also displayed a noticeable decrease during follow-up, the placebo arm had a similar part 1 score compared to baseline at week 4 (time effect, B = −0.48 [−1.69, 0.73], *P* =.44), week 8 (time effect, B = −0.81 [−2.46, 0.83], *P* =.33), and week 12 (time effect, B = −0.56 [−1.76, 0.65], *P* =.37), while the FMT arm showed a significantly additional change in part 1 score at week 4 (group×time effect, B = −2.30 [−4.22, −0.37], *P* <.05) and week 12 (group×time effect, B = −2.19 [−4.27, −0.10] *P* <.05).

In addition to motor dysfunctions, patients with PD often have complaints about nonmotor symptoms such as mood disturbances, cognition disorders, and even sleep disorders, which strongly affect the patient’s quality of life. To investigate whether FMT has a positive impact on the quality of life of patients with PD, we also assessed the MOCA and MMSE scales for cognitive function and GAD-7, GDS-15, and PHQ-9 scales for anxiety and depression.

As Table 3 showed, the MMSE score of participants in FMT had significant additional change compared to the placebo group at week 4 (group×time effect, B = 1.78 [0.45, 3.10], *P* = <.01]). MOCA score in the placebo arm increased significantly at week 4 (time effect, B = 1.82 [0.41, 3.22], *P* <.05), week 8 (time effect, B = 1.93 [0.70, 3.15], *P* <.01), and week 12 (time effect, B = 1.63 [0.55, 2.71], *P* <.01), while the FMT arm had a significantly higher decrease in MOCA score at week 12 compared to the placebo arm (group×time effect, B = 2.26 [0.63, 3.89], *P* <.01), indicating that cognitive function of PD participants might improve better by FMT treatment. However, none of any remarkable changes was observed on the anxiety- or depression-associated scale.

### Gastrointestinal disorders in PD patients were improved by FMT

Apart from PD symptoms, such as bradykinesia, rigidity, resting tremor, and gait impairment, patients with PD often suffer from nonmotor dysfunction, especially gastrointestinal disorders. As a microbiota-modulating therapy, FMT is safe and effective for restoring gut microbiota and treating gastrointestinal symptoms. We performed IBS-SSS, GSRS, and IBS-QoL scales for assessing whether participants benefited from interventions regarding gastrointestinal disorders. Both arms showed improvement in gastrointestinal symptoms.

As Table 3 shows, participants in the placebo group showed a significant decrease in IBS-SSS at week 4 (time effect, B = −36.67 [−61.00, −12.33], *P* <.01), week 8 (time effect, B = −38.89 [−58.48, −19.29], *P* < 0.001), and week 12 (time effect, B = −27.78 [−43.76, −11.80] *P* <.01), while FMT group demonstrated significantly larger decrease compared to the placebo group at week 4 (group×time effect, B = −41.52 [−72.08, −10.96], *P* <.01) and week 12 (group×time effect, B = −40.85 [−71.58, −10.13], *P* <.01). Meanwhile, the GSRS score of the placebo group decreased significantly at week 4 (time effect, B = −1.00 [−1.82, −0.19], *P* <.05) and week 12 (time effect, B = −0.96 [−1.76, −0.17], *P* <.05), while the change in FMT was much larger at week 4 (group×time effect, B = −2.87 [−4.10, −1.64], *P* <.001), week 8 (group×time effect, B = −3.22 [−4.77, −1.68], *P* <.001), and week 12 (group×time effect, B = −3.04 [−4.32, −1.76], *P* <.001). As for IBS-QOL, the placebo arm did not show any significant change during follow-up, but the FMT arm displayed remarkable change compared to the placebo at week 4 (group×time effect, B = −11.48 [−17.52, −5.45], *P* <.001), week 8 (group×time effect, B = −12.52 [−18.85, −6.19], *P* <.001), and week 12 (group×time effect, B = −14.37 [−20.65, −8.09], *P* <.001). The remarkable decrease in the scores of IBS-SSS, GSRS, and IBS-QOL implicated those participants had a significantly better quality of life regarding abdominal pain, flatulence, nausea, etc.

In addition, according to Bristol evaluation, stool characteristics of participants in the placebo arm improved significantly at week 4 (time effect, B = 1.21 [0.37, 2.04], *P* <.01), week 8 (time effect, B = 1.27 [0.41, 2.13], *P* <.01), and week 12 (time effect, B = 1.07 [0.36, 1.78], *P* <.01), but no significant difference was observed when comparing the FMT arm and placebo arm. Also, stool frequency in the FMT arm improved more significantly than placebo group at week 4 (group×time effect, B = 1.37 [0.51, 2.22], *P* <.01), week 8 (group×time effect, B = 1.60 [0.72, 2.49], *P* <.001), and week 12 (group×time effect, B = 1.69 [0.84, 2.54], *P* <.001). This result showed that both arms had desirable outcomes in constipation, especially FMT participants who had a better improvement (Table 3).

### Characteristics of gut microbiota composition in patients with PD differs from healthy donors

To figure out whether the change in gut microbiota is associated with clinical improvement in patients with PD, we performed 16S rRNA sequencing for stool samples from donors (3 times during stool donation), as well as patients at baseline and the 4th and 12th weeks after FMT or placebo intervention.

**Figure 2.**
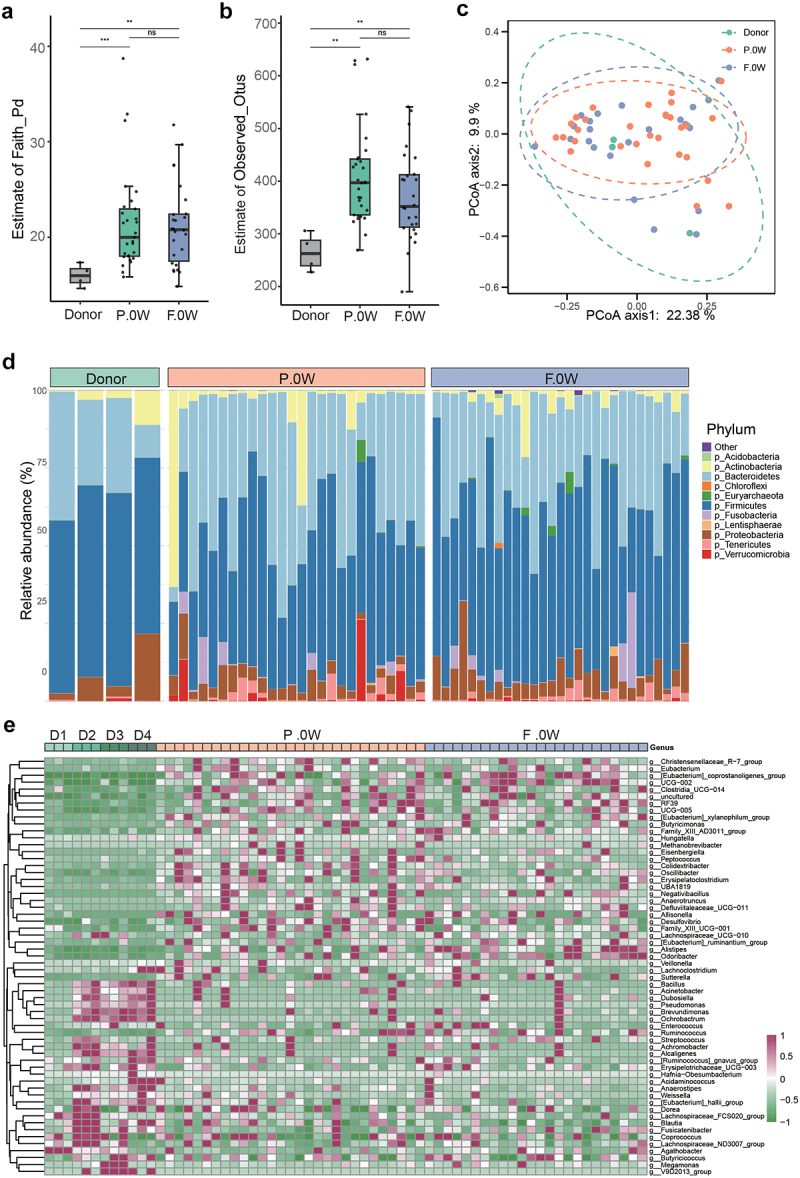
The baseline characteristics of gut microbiota in donors and patients with PD (placebo/FMT group). a-b: α diversity of gut microbiota in different groups according to estimate of Faith_Pd (a) and estimate of Observed_Otus (b) (ns, not significant, ***p <.001; Wilcoxon sum-rank test.); c: Principal coordinate analysis (PCoA) of the microbiota between three groups by R package vegan. (ANOSIM; donor vs. F.0W, R = 0.2055, p = .125; donor vs. P.0W, R = 0.1797, p = .15; F.0W vs. P.0W, R = 0.0041, p = .338); D: bar plots showing the relative abundance of gut microbiota of three groups of individuals at the phylum level, with different colors corresponding to different phyla; E: heat map showing the relative abundance of three groups of individuals at the genus level (samples from the same donor was marked as D1, D2, D3, and D4). Only significantly different genera were shown between donors and patients with PD (R package ALDEx2, we.Ep <.05 and wi.Ep <.05).

Alpha diversity analysis at baseline showed significant differences in PD individuals compared with donors by evaluating the estimate of Faith_Pd or Observed_Otus index (Figure 2a–b). PCoA was performed to investigate the extent of the similarity of the microbial communities in the three cohorts based on unweighted UniFrac distance metrics, and the β diversity of PD patients is heterogeneous compared to donors (Figure 2c). Moreover, the donors, placebo group (P.0 W), and FMT group (F.0 W) displayed different microbial compositions at baseline at both the phylum and genus levels (Figure 2d–e), while no difference of microbiota diversity was observed between the FMT group and the placebo group at baseline (Supplementary Figure S1). The abundance of phylum *Euryarchaeota*, *Firmicutes*, *Lentisphaerae*, and *Tenericutes* was remarkably different between the PD group (P.0 W and F.0 W) and the donor group (Supplementary Table S1). At the genus level, the genera *Blautia*, *Lachnospiraceae_FCS020_group*, *Fusicatenibacter*, *Erysipelotrichaceae_UCG-003*, *[Eubacterium]_hallii_group*, *[Ruminococcus]_gnavus_group*, *Butyricicoccus*, *Lachnoclostridium*, and many other strains were significantly less abundant in the patients with PD, than in donors (Supplementary Table S2).

### Metagenomic sequencing revealed significant differential abundance species between FMT.R and FMT.NR

As our data showed, FMT had a positive effect on clinical outcomes in patients with PD, and remarkable improvement was observed in gastrointestinal disorders. Most patients experienced better bowel movement and had less abdominal pain, flatulence, or other symptoms, and the quality of life was significantly improved in most participants. After 3 consecutive weeks of FMT or placebo intervention, the alpha and beta diversity in gut microbiota did not change significantly (Supplementary Figure S2).

**Figure 3.**
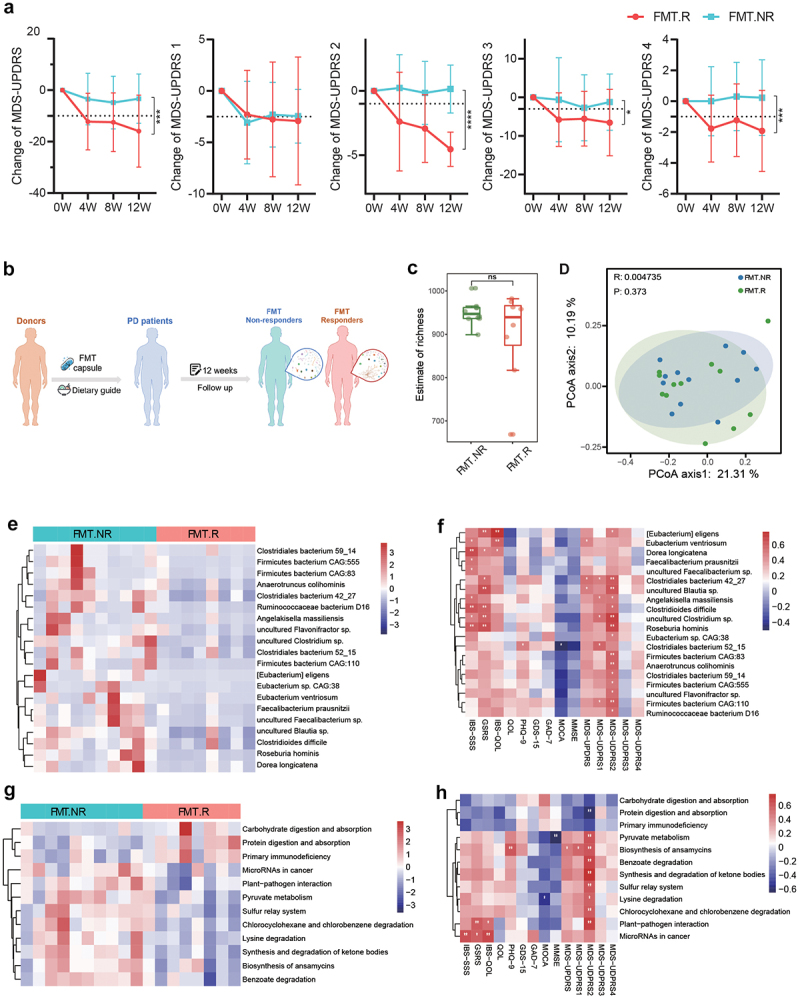
Comparison of gut microbiota between responders and non-responders after FMT treatment. A: patients who underwent FMT treatment had different responses during follow-up according to the MDS-UPDRS score, and the FMT cohort was subdivided into FMT responders (FMT.R, red lines) and FMT non-responders (FMT.NR, cyan lines) based on MDS-UPDRS 2, FMT.R showed a remarkable decrease in MDS-UPDRS total, part 2, part 3, and part 4 score (*: P < .05, **: P < .01, ***: P < .001, ***: P < .0001, generalized estimating equation); B: schematic illustration of subgroup analysis in the FMT arm; C: α-diversity of gut microbiota in FMT.NR and FMT.R subgroups according to estimate of richness; D: Principal coordinates analysis (PCoA) of the microbiota based on the unweighted unifrac distance between FMT.R and FMT.NR subgroups at the end of this trial by R package Vegan; E: metagenomic sequencing analysis showed there are 20 microbial species with significantly different abundance by comparing FMT.NR with FMT.R subgroup; F: the correlation analysis of gut microbiota taxa and clinic outcome of gastrointestinal disorders, PD symptoms, cognition, and depression (*p <.05, **P <.01, Spearman correlation analysis); G: different functional pathways between FMT.NR and FMT.R subgroup; H: the correlation analysis of gut microbiota functional pathways and clinic outcomes. (*p <.05, **P <.01, Spearman correlation analysis).

It is noteworthy that we found that some patients showed obvious improvement in PD symptoms after treatment, while others did not. According to the change in the MDS-UPDRS Part 2 score, which is the Motor Experiences of Daily Living (M-EDL) score, patients were nominated as FMT responders (*n* = 13) and non-responders (*n* = 14), for performing the exploratory ancillary analyses.^24^ As shown in Figure 3a, the MDS-UPDRS scores, including the total score and Part 2, Part 3, and Part 4 scores, of the FMT.R subgroup decreased markedly, while the scores of the FMT.NR subgroup showed only a slight change.

Totally, 3 adverse events occurred in the FMT arm, 1 nausea and 1 flatulence in the responders, and 1 diarrhea in the non-responders (Supplementary table S3). The GEE model was also applied to evaluate the differences between the two subgroups (Supplementary Table S4). As for MDS-UPDRS score, FMT.R had a significant larger decrease in the total score, part 2 score, part 3 and part 4 score at the end of the follow-up, while FMT.NR did not showed any significant change regarding these indicators. Meanwhile, the MDS-UPDRS part 1 score changed remarkably in the FMT.R subgroup, with no additional change between FMT.R and FMT.NR subgroups. These results suggest that subgroups had different responses to PD improvement. Meanwhile, by assessment of IBS-SSS, GSRS, IBS-QOL, and Bristol stool scale, FMT.NR showed significant improvement in bowel movement, but only GSRS score had an additional decrease in FMT.R compared to FMT.NR. The evaluation for cognitive function by MMSE and MOCA scale also showed a significant improvement in the FMT.NR with no additional change in FMT.R, suggesting that patients had a noticeable change in the gastrointestinal disorders as well as cognitive function, regardless of their responses in PD symptoms.

Given that the different responses to FMT treatment might result from individual variations in gut microbiota (Figure 3b), metagenomic sequencing was used to examine the changes in microbial species in the FMT.R and FMT.NR subgroups. At baseline, the microbiota diversity was not significantly different between responders and non-responders, and no taxa was found with significantly different abundance (Supplementary Figure S3). At the 12th week, although the microbiota diversity was not significantly different between the two subgroups (Figure 3c–d), several microbial species were found to be different as shown in Figure 3e, 20 microbial species had notably altered levels of FMT.R individuals compared with FMT.NR. We also noticed that some of these altered species had a strong correlation with the change in patient clinical scores. Specifically, *Eubacterium eligens*, *Eubacterium ventriosum*, *Clostridiales bacterium 42_27*, *uncultured Blautia sp*., *Clostridioides difficile*, *uncultured Clostridium sp.*, and *Roseburia hominis* were positively correlated with gastrointestinal performance and PD symptoms (Figure 3f & Supplementary Figure S4). Additionally, the metagenomic analysis revealed that the two subgroups had different traits in gut microbial functional pathways (Figure 3g). These pathways were also correlated with the change in patient outcomes after FMT intervention, especially the pathway of plant-pathogen interaction, which was positively correlated with the improvement of both gastrointestinal disorders and PD symptoms (Figure 3h & Supplementary Figure S5).

### FMT restored the Bacterial Community Network in FMT-responders of patients with PD

**Figure 4.**
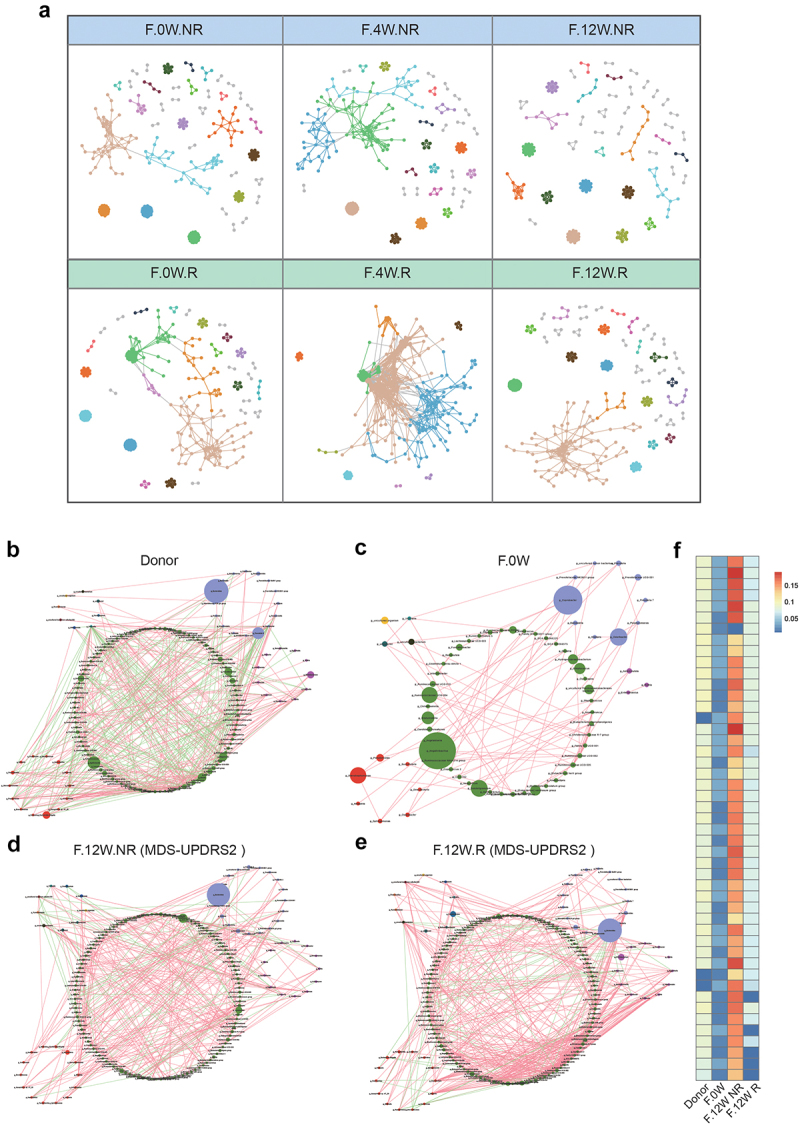
The microbial network analysis in participants after FMT. a: visualization of constructed MENs in FMT.R and FMT.NR subgroups at different times. 18 large modules are shown in different colors, and smaller modules are shown in gray. b-e: comparison of the genera co-occurrence networks between different subgroups. Network plots describing the co-occurrence of bacterial genera in the gut microbiota of donor (b), F.0W (c), F.12W.NR (d), F.12W.R (e) based on the Spearman correlation algorithms (*r* ≥ 0.7, *p* < .05), bacterial genera with at least 0.01% of relative abundance in at least 20% of the samples in each group were plotted. Each node presents a bacterial genus. The node size indicates the relative abundance of each genus per group, and the density of the dashed line represents the Spearman coefficient. Red links stand for positive interactions between nodes, and green links stand for negative interactions; f: discrepancies of the genera co-occurrence networks among groups based on the 16S rRNA data. The centralities (rank of the closeness) and discrepancies of nodes in four subgroups’ co-occurrence networks were counted, respectively.

To determine whether potential changes occurring among bacteria within the gut microbial communities were associated with the different clinical outcomes in FMT.R and FMT.NR subgroups, we constructed two models to investigate the microecological system in the two subgroups. We first constructed 3 time-series molecular ecological networks (MENs) based on the Pearson correlations of log-transformed ASV abundances. As shown in Figure 4a, the complexity of microecological network features in FMT.R increased prominently once FMT treatment was started and gradually returned to the original status. Details of the network topological attributes are listed in Supplementary Table S5. However, we did not observe a significant change in the FMT.NR subgroup. Next, to further confirm the alteration of the microbial community between two subgroups, we constructed co-occurrence networks of genera from each subgroup. Consistent with our prediction, the microbial network of participants after FMT intervention was more complicated than the baseline (Figure 4b–f). Similar to the results based on MENs, the correlation among gut microbes further indicated that responders had more complex microecological structures than non-responders (Figure 4d–e). The microbial network in the FMT.R subgroup at the end of the trial showed greater similarity to donors in comparison to FMT.NR. Next, to quantify the difference in the donors, patients with PD before FMT, FMT.R, and FMT.NR, we counted the number of edges (connections) and the centrality of the nodes (genera) in the four microbial networks. As shown in Figure 4f, the results showed that there were significant differences in the closeness and characteristic carriers of the shared genus between patients and donors, but no obvious difference between the FMT.R and the donors was observed. In summary, these findings suggested that FMT may alleviate the clinical symptoms of patients with PD by strengthening the correlation between the microbial genera and improving the microecological situation of patients with PD.

## Discussion

Increasing reports showed that dysbiosis in the gut microecological system promotes altered host-microbial interactions and might aggravate disease progression in many neurological conditions, such as PD, autism spectrum disorders, Alzheimer’s disease, multiple system atrophy, and amyotrophic lateral sclerosis.^25^ This evidence urged us to figure out whether medications that focus on the gut, especially FMT, could benefit the pharmaceutical treatment for PD patients through reconstructing the gut micro-environment, as our previous data already revealed that FMT could significantly improve the behavior and cognitive function in autistic children through the gut-brain axis,^26^ and several clinic reports verified that FMT had a positive impact on improving symptoms in PD patients.^21–23,27^

In the present work, we carried out an RCT study on the effects of FMT on patients with PD. We used freeze-dried fecal microbiota powder capsules, this preparation is much more acceptable and accessible for patients than conventional endoscopic routes such as colonoscopy or gastrointestinal tube, which require advanced endoscopists or radiological equipment, capsule preparation allows patients get FMT at home or through distant delivery. Herein, we demonstrated that oral FMT significantly improved the effect of conventional PD medications on PD-associated symptoms. Before the intervention started, participants in FMT and placebo group demonstrated similar clinical characteristics regarding the evaluation for PD, at the end of this trial, FMT participants demonstrated a significantly greater change in MDS-UPDRS total score, showing that PD symptoms were alleviated.

It has been generally realized that the gut microbiota is crucial in the physiological processes of CNS, including neurogenesis, myelination, microglial activation, etc. Similar cross talk exists between the CNS and gut microbiota in the progression of neurodegenerative disease, which involves not only motor symptoms but also non-motor symptoms (ie, cognition, mood, constipation, and many other aspects). Our data showed that non-motor experiences in the daily life of patients (assessed by the MDS-UPDRS part 1 scale) were significantly improved by FMT, which supports our previous hypothesis that FMT is efficient in improving the quality of life for PD patients. Previous report showed that FMT significantly improved constipation in PD patients,^28^ in our work, the improvement in non-motor aspects not only included gastrointestinal symptoms but also cognitive function. The participants after FMT treatment showed better outcomes in cognitive function through MOCA assessment. During this trial, we observed a significant increase in MOCA score in the placebo participants at weeks 4, 8, and 12; moreover, FMT participants demonstrated a more significant increase in MOCA score at week 12. In cognitive assessment, re-testing bias might lead to the improvement in the score after several rounds of follow-up interviews, it is hard to tell whether the improvement in MOCA score in placebo participants was induced by their daily medication or by re-testing bias, but it is identical that FMT participants showed a greater improvement than the placebo group in MOCA. This implies that FMT treatment might be helpful in cognitive function repair for PD patients, and we believe this result is valid due to the evaluation by MOCA as well as MDS-UPDRS part 1 score.

In addition, during the follow-up, a high placebo response to gastrointestinal symptoms was observed, which was in accordance with numerous reports of interventional studies of patients with gastrointestinal disease. Patients with functional gastrointestinal disorders tend to be sensitive to placebo treatment.^29^ Moreover, dietary guidance is essential in almost all interventional clinical trials for gastrointestinal disorders. Unlike irregular dietary and nutrient uptake, a healthy diet pattern is beneficial for somatic symptom management.^30^

Specifically, compared with the placebo arm, the FMT arm had a more significant decrease in MDS-UPDRS part 1 score at week 4 and week 12, showing that patients had a significantly better quality of life. Common knowledge on Parkinson’s Disease often focusses on motor dysfunction, but the poor life quality that greatly affects patients’ daily life. Moreover, the associated heavy burden for caregivers and social cost has now become an attractive issue in PD treatment. Since the MDS-UPDRS part 2 score represents the motor experiences in daily life, and the change of it could be an indicator for evaluating clinical improvement in PD patients^24^, we performed a post-hoc analysis by subgrouping participants in the FMT arm into FMT.R and FMT.NR according to the change of part 2 score at week 12. The FMT.R had significantly lower total scores and subscores (except for part 1) of MDS-UPDRS at week 12, while all participants showed significantly decreased part 1 score, at the same time, FMT.NR had a remarkable improvement in gastroenterology-associated evaluations and cognitive function, and there is no significant difference between FMT.R and FMT.NR in this aspect. Consistent with our prediction, FMT.R had a significantly lower GSRS at week 12. These results further confirmed that FMT induced improvement in non-motor symptoms for PD patients, regardless of their responses in motor function. As for those FMT.R participants whose motor functions improved well, their improvement in the above-mentioned aspects is also comparable to FMT.NR.

Dysbiosis in the gut microecological system promotes altered host-microbial interactions and might aggravate the disease progression in many neurological dysfunctions like PD, autism spectrum disorders, Alzheimer’s disease, multiple system atrophy, amyotrophic lateral sclerosis.^26,31–35^ As our data shown, compared with healthy donors, the top 5 changed bacteria at the genus level in patients with PD were the *Blautia* (decreased), *Lachnospiraceae_FCS020_group* (decreased), *Fusicatenibacter* (decreased), *Erysipelotrichaceae_UCG-003* (decreased), and *Enterococcus* (increased). These genera are closely related to several neurological disorders, including PD, cognitive impairment, dementia, major depressive disorder, and multiple sclerosis.^36–39^

Based on these results, we hypothesize that the effectiveness of FMT on PD symptoms is contributed by the alteration of gut microbiota. Interestingly, we observed distinct responses from participants in the FMT arm, where some showed significant improvement in symptoms while others did not. We speculated this outcome might be related to the efficacy of FMT in altering the gut microbiota as well. This hypothesis was then confirmed by subgroup comparison between responders and non-responders to FMT therapy. Metagenomic analysis reveals that some butyrate-produced bacteria from the phylum Firmicutes, such as *Eubacterium eligens*, *Eubacterium ventriosum*, *Clostridiales bacterium 42_27*, *uncultured Blautia sp*., *Clostridioides difficile*, *uncultured Clostridium sp*., and *Roseburia hominis* are enriched in FMT.R group compared with FMT.NR group. Moreover, these species showed positive correlations with the improvement of gastrointestinal function as well as PD symptoms. Consistent with the previous report, the genera, *Blautia* and *Roseburia*, have been identified as markedly decreased groups in PD patients compared with healthy controls.^40^ The taxa *Blautia* and *Roseburia* belong to the family *Lachnospiraceae*, the two butyrate-producing taxa are also found to be decreased in other neurological disorders,^41^ butyrate and other SCFAs are the pivotal energy source for gut epithelial, butyrate can affect the enteric nervous system (ENS) through anti-inflammatory properties and promoting microglia development, and potentially affect epigenesis in the CNS. Moreover, a recent report by Herbert L. DuPont and colleagues explored the efficacy of FMT for treating PD patients associated with constipation and also showed that *Roseburia* became dominant in the gut microbiota in long-term follow-up, which strongly inspires us that these taxa certainly play a pivotal role in the microbiota-host interaction during the progress in PD.^27^ However, the recent trial reported that *Blautia* and *Eubacteria* transiently decreased in PD patients after FMT treatment, which is inconsistent with our result, hence, these candidate species still need to be further explored in their underlying roles in the gut-brain axis in PD.

On the other hand, we observed that the FMT.R group had a more closely connected microbiota network compared with FMT.NR group. Similar outcomes were also reported in other studies. For example, Holvoet et al. reported that FMT induced different responses in patients with irritable bowel syndrome due to different features in gut microbiota.^42^ In this trial, we observed that the correlation between gut microbes and microecological complexity in FMT responders was higher than in non-responders and those who received a placebo, which might lead to participants having variable responses under FMT treatment. It has been reported that gut microbiota interventions could also affect the availability and pharmacokinetics of medication used in neurological disorders, which may lead to increased efficacy and a different side effect profile.^43^ Moreover, gut microbiota has been confirmed as a biomarker for clinical outcomes in immunotherapy.^44^ Further exploration is needed to determine whether FMT could enhance the efficacy of PD medicine or influence endocrine, metabolic, or other pathways through the rejuvenation of gut microbiota.

This study showed a promising result for FMT in improving the effectiveness of conventional PD medication. However, there are certain major limitations in this study, which might reduce the generalizability of this trial. The sample size is small and investigators were not masked from the participants’ allocation, which would introduce an imbalance in demographic or clinical characteristics in groups even though randomization was carried out. In addition, the comparability of the total MDS-UPDRS score for the two arms is marginal at baseline (with a p-value of 0.06), indicating a possible imbalance of disease severity between the two groups, meanwhile, there was also significant difference for MDS-UPDRS part 2 score at baseline, we speculate that this might be led by the small sample size of our trial, which is a major limitation of this work.

To present the result more objectively, the unstructured correlation has been used in generalized estimating equations to assess the interaction of group and time, then minimize the re-testing bias, but there is still some bias that we cannot avoid in interpreting this. A larger cohort with a double- or triple-blind design of FMT on improving PD medication is still needed for further exploring the effectiveness. Another major limitation of this study is that 3 months of follow-up for PD patients is relatively short, our study only demonstrated the short-term effectiveness and safety in patients, based on current data, we may have a better understanding in future exploration of the efficacy of FMT on PD patients.

Our clinical data showed that dietary guidance might also improve PD-associated gastrointestinal dysfunction, motor disorders, cognition, and mood disorders, which implied that the placebo might serve as a helpful treatment. This trial was performed in southwest China, where irritating food (mostly greasy and spicy food) is common in people’s daily lives, after enrollment, patients were asked to follow a specific regular and healthy diet, with limited protein intake and high-fiber diet, which may affect their composition of gut microbiota. Unfortunately, we did not record the dietary information during the intervention and follow-up, so we can only assume that dietary guidance affected gastrointestinal disorders and the quality of life of participants. In this study, we did not assess whether geological factors had an impact on the therapeutic outcomes in patients with PD, so further multicenter studies involving patients from different regions would be beneficial. Another major limitation of this study is that we did not perform a longer follow-up on participants. The current data only revealed a relatively short outcome in PD patients.

In conclusion, this trial demonstrated that the oral FMT capsule was well-tolerated in patients with PD, it was effective in restoring the gut micro-ecology in PD patients, improving gastrointestinal symptoms and quality of life in PD patients, and was promising in improving the efficacy of conventional treatment for PD symptoms and cognitive function. We provided evidence that oral FMT capsules can be safely and feasibly engrafted in these patients, demonstrating the promising potential of FMT in improving current PD medications. Our study also suggested that gut microbiota could serve as a biomarker to predict the response to FMT intervention in PD.

## Materials and methods

### Study design

This randomized, placebo-controlled trial was carried out at the Department of Neurology, Army Medical University Southwest Hospital, from February 2019 to December 2019. The study was conducted in accordance with the guidelines and principles of the Declaration of Helsinki, approved by the Administrative Panel for Medical Research on Human Subjects (the Ethics Committee) of Army Medical University Southwest Hospital in Chongqing, China, and the trial registration number was ChiCTR1900021405 (http://www.chictr.org.cn/). Full protocol is presented in the Supplementary materials.

### Participants

Subjects aged 30 to 85 who were diagnosed with mild to moderate PD (Hoehn-Yahr stage 1 to 3) according to the diagnostic criteria for PD in China (2016 edition) were enrolled. Detailed inclusion criteria are as follows: 1) male and female aged 30 to 85 years, 2) patients with early Parkinson’s disease are diagnosed as having H-Y 1 ~ 3 grade of Parkinson’s disease in accordance with the diagnostic criteria for Parkinson’s disease in China (2016 edition), 3) with no specific difficulty in communication, 4) sign the informed consent and agree to participate in this study. Detailed exclusion criteria are as follows: 1) complicated with other serious diseases of the heart, liver, kidney, respiratory, digestive, blood and endocrine systems, 2) combined with intestinal double infection, such as CDI, ehec, salmonella, shigella, campylobacter, plague, and cytomegalovirus, 3) liver function was significantly abnormal or had the following liver disease history: AST or ALT was twice higher than the upper limit of normal value (AST: 59 U/L, ALT: 50 U/L), history of liver cirrhosis, hepatic encephalopathy, history of esophageal varicose veins or portal shunt, 4) with evidence of renal damage or the following renal disease history: serum creatinine is 1.5 times higher than the upper limit of normal value; The history of dialysis; Or a history of nephrotic syndrome, 5) patients with various acute infections, tumors and severe arrhythmias, mental disorders, drug or alcohol addiction, 6) pregnant or lactating women, 7) have used antibiotics or microecological agents in the past 4 weeks, 8) patients with allergy or contraindications to “enterobacteria capsule” used in this study, 9) clinical researchers with other related Parkinson’s disease were being conducted at the time of enrollment or within 3 months before enrollment, 10) it is difficult to complete the interview, or various factors affecting the compliance.

According to current reports, the therapeutic efficacy of PD patients is about 48%, and the estimated efficacy of FMT on PD patients is 80%. Assuming the allocation of 1:1, type 1 error of 0.05 (α), and power of 0.9 (1-β), based on former reports about conventional treatment and FMT on Parkinson’s disease, 56 participants were enrolled assuming a 10% dropout rate to detect a minimum difference in MDS-UPDRS scores between the FMT and placebo groups.

### Randomization and masking

All patients were recruited by physicians and trial investigators at the Department of Neurology, Southwest Hospital, Chongqing. The enrolled participants were randomly assigned to the FMT group and the placebo group in a 1:1 ratio by using computer-generated random numbers. Participants were assigned to FMT or placebo group by trial investigators and remained masked to the allocation of treatment, while investigators and clinicians were not. The appearance of FMT and placebo capsules was indistinguishable, and all capsules were taken at the hospital to ensure that participants were unaware of the content.

### Interventions

We selected 4 stool donors according to strict screening based on previously reported criteria.^45^ Stool from these 4 donors were used for this trial and other clinical use at the same time. For stool donation for this trial, each donor provided stools for making FMT capsules for about 7 patients, and each patient in the FMT group was given 16 FMT capsules at each time, which are made from approximately 50 g of donated stool. Donor-recipient pairing was not performed in this study, and all patients received FMT from a random donor each time. FMT capsules (patent NO.: ZL201510304041.4) were prepared as previously reported.^45^

Sixteen capsules of FMT or placebo were taken orally by participants at once in the morning on an empty stomach, all subjects received either FMT capsules orally or placebo capsules once a week for 3 consecutive weeks. The appearance of FMT and placebo capsules was indistinguishable, and all capsules were taken at the hospital to ensure that participants were unaware of the content. Subjects in both groups maintained the original medication for PD before enrollment and maintained the regimen during the whole trial. Moreover, medical guidance helped them to maintain a healthy diet and rest schedule, details of dietary guidance and precautious of FMT are presented in the Supplementary Appendix.

### Outcomes

The primary outcome was the MDS-UPDRS score of the subjects at week 12. Secondary outcomes were the MDS-UPDRS score at weeks 0, 4, and 8, safety (adverse effects) at weeks 0, 4, 8, and 12, and evaluation for gastrointestinal disorders (including the IBS-SSS, GSRS, Bristol stool form scale, and IBS-QOL scale scores) and evaluation for mental health (including the PHQ-9 scale, GDS-15 scale, GAD-7 scale, Montreal Cognitive Assessment, Mini-mental State Examination scores) at weeks 0, 4 8 and 12, and alterations in gut microbiota at weeks 0, 4 and 12, including alpha diversity (Shannon index) and beta diversity (PCoA) at the genus level, microbial community composition among donors, the FMT group, and the placebo group.

### Subgroup analysis

To determine the connection between gut microbiota and PD symptoms after FMT treatment, we further studied the effect of FMT on PD by dividing participants in the FMT group into responder and non-responder subgroups, as the exploratory and ancillary analyses. According to the change in the MDS-UPDRS Part 2 score, which is the Motor Experiences of Daily Living (M-EDL) score used for measuring the changes in the level of disability of patients with PD, patients with a decrease in MDS-UPDRS 2 score larger than 3.05 were considered FMT responders, while the rest were deemed non-responders based on previous reports on minimal clinically important differences in MDS-UPDRS 2 scores.^24^

### DNA isolation and 16S rRNA gene sequencing

Feces from participants and donors were collected at the hospital or home according to the collection instructions and delivered immediately on dry ice. Specifically, each donor provided stool samples for 6 weeks for FMT capsule manufacturing, and fecal samples from donors were collected at weeks 0, 3, and 6. Meanwhile, fecal samples of patients from both groups were collected at baseline and weeks 4 and 12 during follow-up. In total, 0.18–0.22 g stool samples were used to extract total bacterial genomic DNA following the protocol of the DNA extraction kit (#DP328, Tiangen Company, Beijing, China). The concentration and purity of the extracted bacterial DNA were detected using the Qubit 2.0 Fluorometer (Thermo Scientific, USA). The 16S rRNA gene of the V4 region-specific primers was 515F GTGCCAGCMGCCGCGGTAA and 806 R GGACTACHVGGGTWTCTAAT. Sequencing libraries were generated using an Illumina TruSeq DNA PCR-Free Library Preparation Kit (Illumina, USA) following the manufacturer’s recommendations, and index codes were added. The library was sequenced on an Illumina HiSeq platform, and 250 bp paired-end reads were generated (Novogene, China). The mean of Raw PE reads is 91,819.

Microbiome bioinformatics was performed with the QIIME2 software package (Quantitative Insights Into Microbial Ecology)11.^46^ Raw sequence data were demultiplexed and quality filtered using the q2-demux plugin followed by denoising with DADA2. All amplicon sequence variants (ASVs) were aligned and used to construct a phylogeny with fasttree2.^47^ Principle coordinate analysis (PCoA) was performed using the vegan v2.5–7 R package. For the diversity of donors, the mean alpha and beta diversity of each donor was calculated based on the 3 samples of each donor. Gene functions were predicted by PICRUSt2 v2.4.1 and were calibrated using Meta-Apo based on the Metacyc database.^48^

### Shotgun sequencing for metagenomics

Approximately 2 μg DNA per sample was prepared. Sequence libraries were generated using the NEBNext Ultra DNA Library Prep Kit for Illumina (NEB, USA). The libraries were sequenced on the Illumina NovaSeq 6000 platform (insert size 350 bp, read length 150 bp) at Novogene Bioinformatics Technology Co., Ltd. (Tianjin, China).

Metagenomics raw data is about 6 G each sample. Raw sequence reads were trimmed using Trimmomatic v0.39 to remove adapters and low-quality regions and then removed from contaminating human reads using Bowtie2 v2.4.2 (Reference database: GRCh38).^49^ The taxonomic composition was profiled using the default parameters of MetaPhlAn3 v3.0.9.^50^ The functional gene pathway was profiled using the default settings of HUMAnN3 v3.0.0.alpha.3.^51^ For the correction of statistics, Benjamin Hochberg’s adjustment was applied in between-group comparison and correlation analysis.

### Co-occurrence network analysis

Microbial network analysis was performed as previously reported.^52^ To understand the correlations among different genera, we constructed a co-occurrence network based on the 16S rRNA sequencing data. The bacterial correlations in the FMT group at week 0 (F.0 W), FMT non-responders at week 12 (F12 W. NR), and FMT non-responders at week 12 (F12 W. R) were analyzed according to the relative abundance of each genus using Spearman’s correlation coefficient to construct the cooccurrence network. The significantly correlated genus (false discovery rate < 0.05, rho ≥ 0.7) was visualized by Cytoscape v3.8.2.^53^ The closeness centrality of the shared nodes was calculated by the igraph R package. Only genera that existed in at least 20% of the samples were included in the network analysis.

### Correlation analysis of genus and clinical outcomes

To determine the association between genus and clinical outcomes in participants, we performed a correlation analysis using Spearman’s correlations (*P* value < 0.05) in the psych R package. The results were visualized by pheatmap R packages.

### Statistical analysis

Descriptive statistics were performed to demonstrate the demographic features, health conditions, and clinic manifestations at baseline and each time point during follow-up, continuous data were presented as mean(sd), categorical data were presented as median (IQR), and binary data were presented as n (%).

To analyze the differences in the subject’s characteristics between the placebo and FMT groups after the intervention, generalized estimating equation (GEE) models were used to analyze the primary and secondary outcomes (including the total score of MDS-UPDRS and Part 1, Part 2, Part 3, Part4, and the scores of IBS-SSS, GSRS, IBS-QOL, MMSE, MOCA, PHQ-9, GDS-15, GAD-7, Bristol stool scale, and stool frequency). For the GEE analysis in this work, time was set as within-subject variables, an independent correlation matrix with robust SEs was used to account for the correlation between multiple responses for the same participants. A linear regression for score outcomes (MDS-UPDRS total score, MDS-UPDRS part1/2/3/4, GSRS, IBS-SSS, IBS-QOL, MMSE, MOCA, PHQ9, GDS-15, and GAD7), was used to estimate mean differences, ordinal logistic regression for ordinal outcomes (Bristol and stool frequency). Models treated allocated intervention (Group) and time point (Time) as main effects, and Group*Time as interact effect. Time effect was the difference by comparing FMT (or placebo) arm at each timepoint and the baseline. Group×time effect was time-by-group interaction, representing the difference of the time effect between two arms at each timepoint. Statistical analysis was performed using SPSS statistical software, version 23·0 (IBM Corporation). All statistical tests were 2-tailed with a 5% level of statistical significance.

## Supplementary Material

Supplemental Material

## Data Availability

All sequencing data generated in the preparation of this manuscript have been deposited in the China National GeneBank DataBase (CNGBdb, https://db.cngb.org/) with accession number CNP0002130 for 16S sequencing, and CNP0004940 for metagenomic sequencing. Anonymized data will be made available upon reasonable request to the chief investigator. Proposals will be reviewed and approved by the sponsor, chief investigator, and collaborators based on scientific merit. After approval of a proposal or request, data can be shared through a secure online platform after signing a data access agreement.
