# Supplementary material for: Efficacy of fecal microbiota transplantation in patients with Parkinson’s disease: clinical trial results from a randomized, placebo-controlled design
Source: Gut Microbes. 2023 Dec 6;15(2):2284247. doi: 10.1080/19490976.2023.2284247 (PMC10841011; doi:10.1080/19490976.2023.2284247)
Supplement: Supplemental Material [file KGMI_A_2284247_SM4073.zip › supplementary appendix_dietary guide.docx]

**Diet guidance and precautions for FMT**

1.Amoxicillin, cephalosporins and other antibiotics are prohibited.

2.Avoid alcohol, coffee, tea, carbonated drinks.

3.Do not overeat, Life should be orderly and meals should be regular.

4.Do not eat cold, spicy, greasy, pickled, smoked and baked foods.

5.Better to eat light, non-irritating, less residue, soft, easily digestible food.

6.Drinking more water. The combination of water and intestinal fiber can increase the volume of stool and facilitate defecation.

7. Eat less gas-producing foods, such as soybeans, sweet potatoes, apples, grapes, etc., which are easily decomposed by bacteria, resulting in flatulence, abdominal pain and other symptoms.

8.Constipation patients should avoid refined foods, increase the intake of dietary fiber, eat more fruits and vegetables and grains, such as leeks, celery, yams and grains and beans.

9. In life, you should eat less foods rich in sorbitol and fructose, such as apples, watermelons, grapes, cream products, etc.

10. Eat less high-protein food, suchs as egge, fish, chicken, etc.

Notes:

1. Capsules need to be taken at empty stomach in the morning, one patch (16 capsules) at a time, 1 times/week;

2. Defrost at room temperature 30 minutes before taking and consume within 30 minutes to 1 hour after thawing
