# Supplementary material for: Efficacy of fecal microbiota transplantation in patients with Parkinson’s disease: clinical trial results from a randomized, placebo-controlled design
Source: Gut Microbes. 2023 Dec 6;15(2):2284247. doi: 10.1080/19490976.2023.2284247 (PMC10841011; doi:10.1080/19490976.2023.2284247)
Supplement: Supplemental Material [file KGMI_A_2284247_SM4073.zip › Supplementary figures (1).docx]

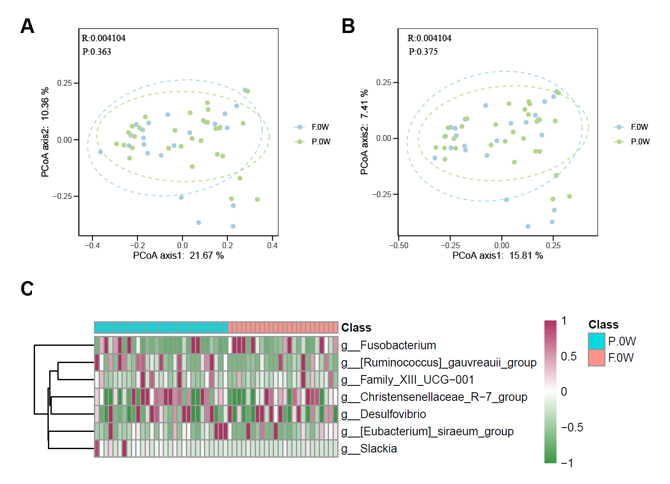


**Supplementary figure 1.** A-B: The gut microbiota β diversity of the participants in FMT and placebo groups at baseline by the Bray-curtis (A) and Jaccard (B); C: The pheatmap of different taxa of the FMT group and the placebo group at basline.


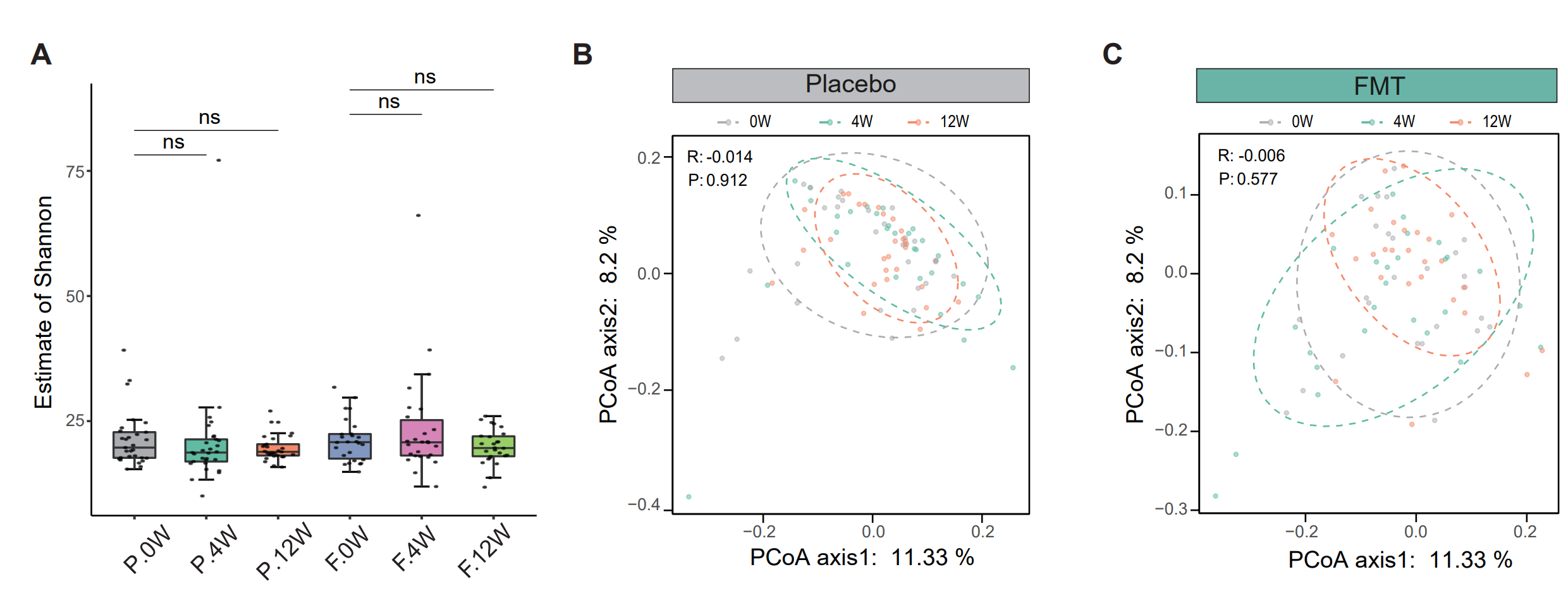


**Supplementary figure 2.** A: The gut microbiota α diversity of the participants in FMT and placebo groups by the estimate of Shannon index; B-C: The gut microbiota β diversity of the participants in placebo (B) and FMT (C) groups by PCoA.


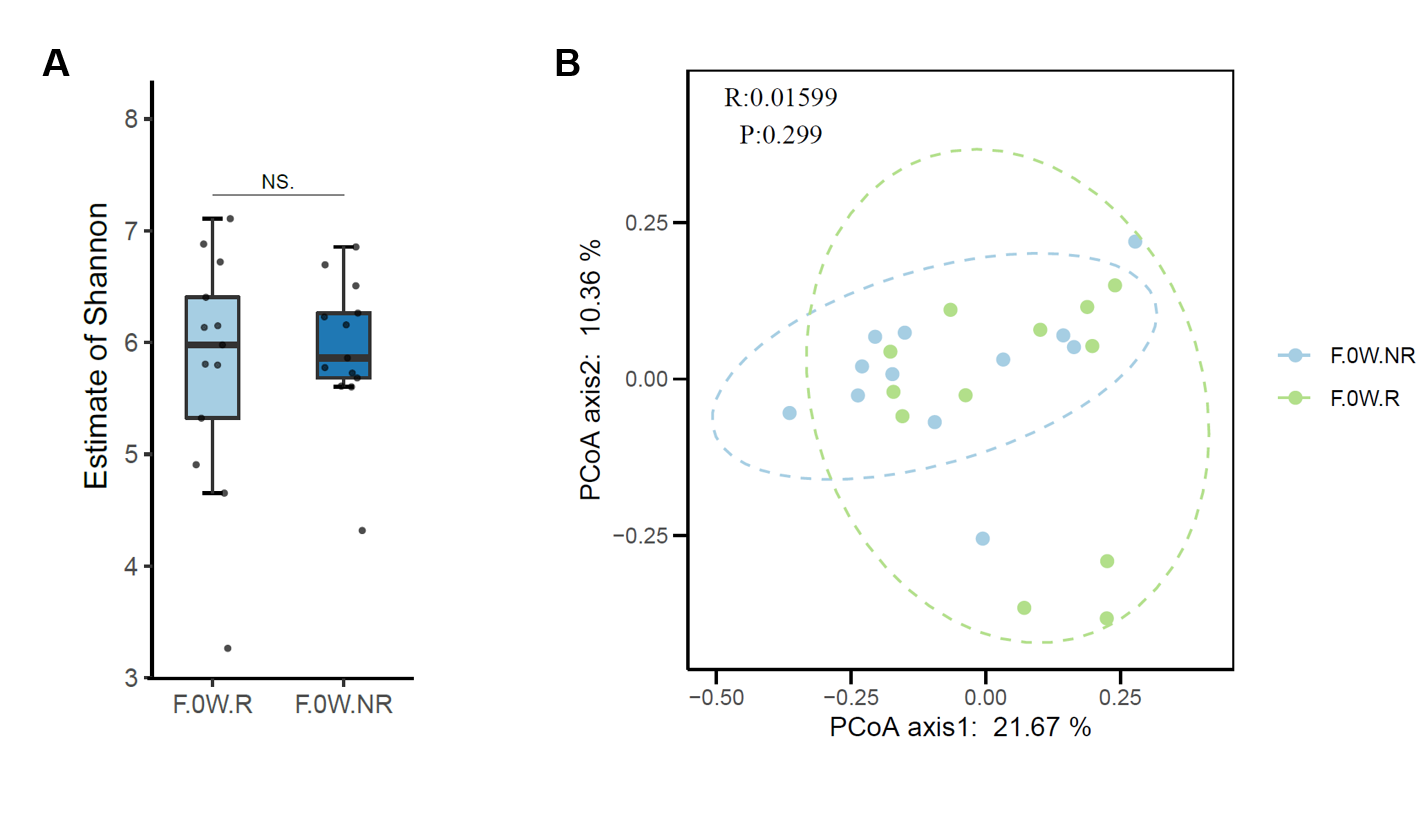


**Supplementary figure 3.** A: The alpha diversity (shannon index) of responder and non-responders in the FMT arm; B: The beta diversity (Bray-curtis) of responder and non-responders in the FMT arm.


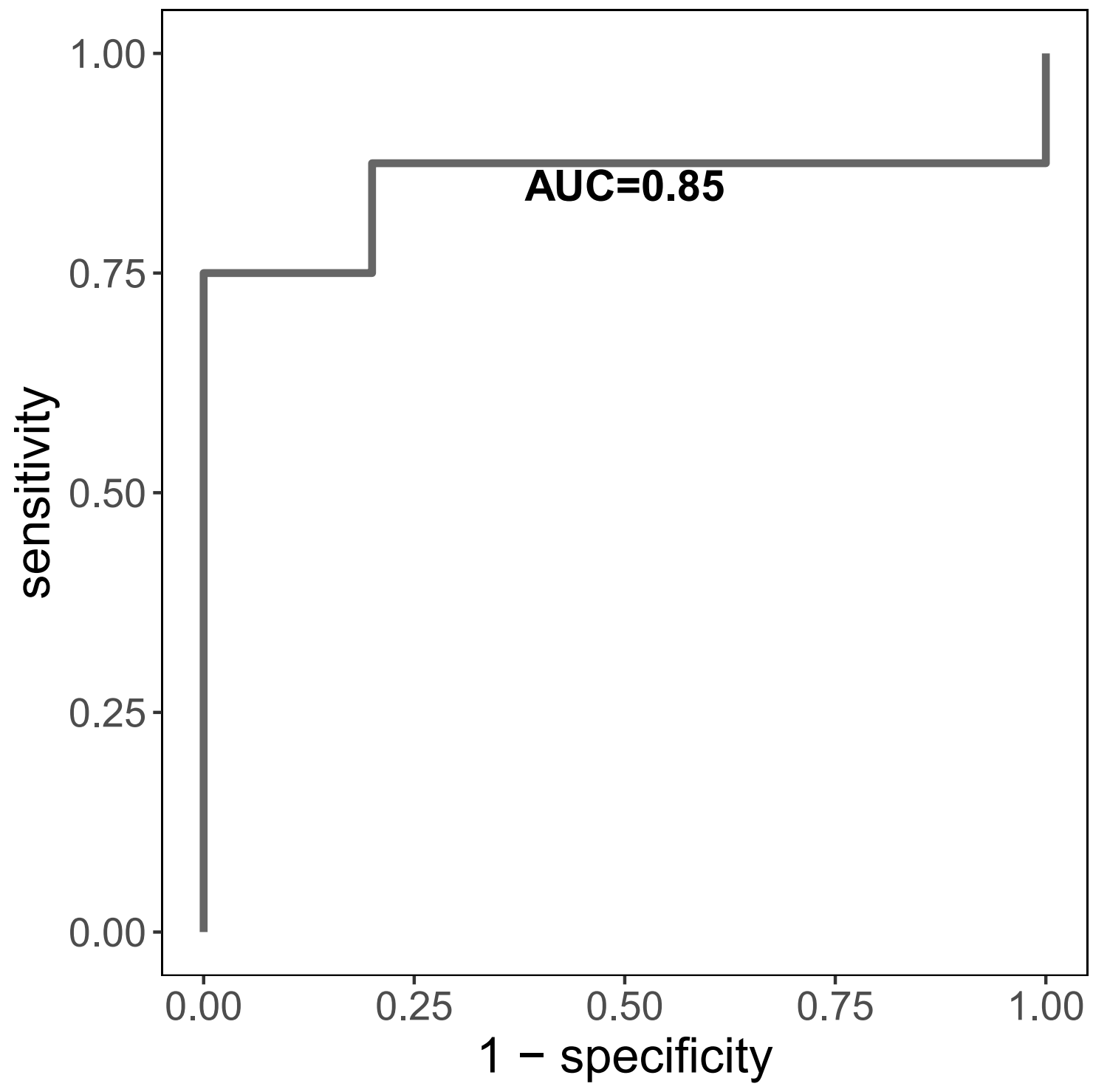


**Supplementary figure 4.** The ROC plot of correlation analysis between gut microbiota species and clinic outcomes.


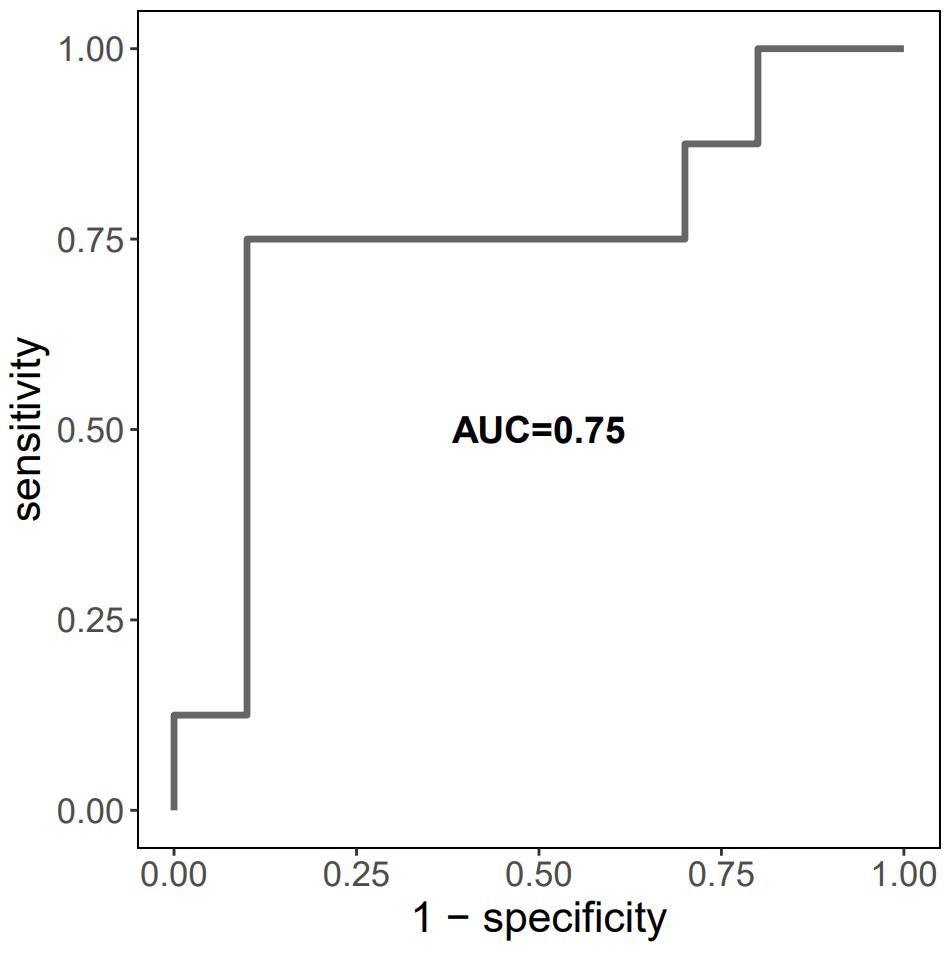


**Supplementary figure 5.** The ROC plot of correlation analysis between gut microbiota functional pathways and clinic outcomes.
