# Supplementary material for: Efficacy of fecal microbiota transplantation in patients with Parkinson’s disease: clinical trial results from a randomized, placebo-controlled design
Source: Gut Microbes. 2023 Dec 6;15(2):2284247. doi: 10.1080/19490976.2023.2284247 (PMC10841011; doi:10.1080/19490976.2023.2284247)
Supplement: Supplemental Material [file KGMI_A_2284247_SM4073.zip › Supplementary protocol.docx]

**Table of Content**

[Abbreviation List 3](#_Toc59181957)

[Trial Procedure 4](#_Toc59181958)

[Trial Protocol 10](#_Toc59181959)

[1. Background and Significance 10](#_Toc59181960)

[2. Objectives of Study 14](#_Toc59181961)

[3. Study Design 14](#_Toc59181962)

[4. Fecal Microbiota Transplantation (FMT) 15](#_Toc59181963)

[5. Participants: 16](#_Toc59181964)

[5.1 Inclusion criteria: 16](#_Toc59181965)

[5.2 Exclusion criteria: 16](#_Toc59181966)

[5.3 Withdrawal criteria 17](#_Toc59181967)

[6. Trial Procedure and Outcomes 18](#_Toc59181968)

[6.1 Trial Procedure 18](#_Toc59181969)

[6.1.1 Wash out ( -1~0 week) 18](#_Toc59181970)

[6.1.2 Baseline information (0 week) 18](#_Toc59181971)

[6.1.3 Interventions (1-3 week): 21](#_Toc59181972)

[6.1.4 End point (3 month) 21](#_Toc59181973)

[6.2 Follow up 21](#_Toc59181974)

[7. Outcomes: 29](#_Toc59181975)

[7.1 Primary outcomes: 29](#_Toc59181976)

[7.2 Secondary outcomes: 29](#_Toc59181977)

[8. Adverse events: 29](#_Toc59181978)

[8.1 Adverse events: 29](#_Toc59181979)

[8.2 Serious Adverse Events (SAE): 32](#_Toc59181980)

[9. Sample Collection 33](#_Toc59181981)

[Collection of stools 33](#_Toc59181982)

[10. Participant management 33](#_Toc59181983)

[10.1 Drop out 33](#_Toc59181984)

[10.1.1 Criteria for drop out 33](#_Toc59181985)

[10.2 Concomitant medication or treatment 34](#_Toc59181986)

[10.2.1 Prohibited medication or treatment 34](#_Toc59181987)

[10.2.2 Allowed medication or treatment 34](#_Toc59181988)

[11. Data management and statistical analysis 34](#_Toc59181989)

[11.1 Data management 34](#_Toc59181990)

[12. Preservation of data 34](#_Toc59181991)

[13. Ethics 34](#_Toc59181992)

[13.1 Ethics Committee 35](#_Toc59181993)

[13.2 Informed consent 35](#_Toc59181994)

[14. Responsibilities and Regulations 35](#_Toc59181995)

[14.1 Investigators 35](#_Toc59181996)

[14.1.1 The investigator or designated representative must provide detailed information about the trial 35](#_Toc59181997)

[14.1.2 Informed consent should be obtained after full and detailed explanations 36](#_Toc59181998)

[14.1.3 Protection of participant’s privacy 36](#_Toc59181999)

[14.1.4 Protection of participant’s safety 37](#_Toc59182000)

[14.1.5 Guarantee of trial quality 37](#_Toc59182001)

[14.2 Cooperator 38](#_Toc59182002)

[14.3 Supervisor 38](#_Toc59182003)

[14.4 Protocol modification 39](#_Toc59182004)

[14.5 Usage of information related to the trial 39](#_Toc59182005)

[Reference 40](#_Toc59182006)

Abbreviation List

| AE | Adverse events |
| --- | --- |
| ALT | Alanine aminotransferase |
| ALP | Alkaline phosphatase |
| AST | Aspartic acid amino transferase |
| BUN | Urea nitrogen |
| CRF | Case report form |
| CRP | C-reactive protein |
| Cys C | The elf inhibition C |
| GCP | Good clinical practice |
| GGT | γ- glutamyl transpeptidase |
| Hb | Hemoglobin |
| Hcy | Homocysteine |
| LY | Lymphocyte count |
| MID | Neutrophil percentage |
| MONO | Monocyte count |
| PLT | Platelet count |
| RBC | Reed blood cell count |
| SAE | Severe adverse events |
| Scr | Creatine |
| TBIL | Total bilirubin |
| UA | Uric acid |
| WBC | White blood cell count |

Trial Procedure

|  | V0 | V1 | V2 | V3 | V4 | V5 | V6 |
| --- | --- | --- | --- | --- | --- | --- | --- |
| Time relative to V1 | -1 wekk | 0 week | 1 week | 2 week | 4 week | 8 week | 12 week |
| Baseline information |  |  |  |  |  |  |  |
| Informed consent | **√** |  |  |  |  |  |  |
| Medical history | **√** |  |  |  |  |  |  |
| Concomitant disease and medication | **√** | **√** |  |  |  |  |  |
| Inclusion/exclusion criteria | **√** | **√** |  |  |  |  |  |
| Baseline questionnaire |  | **√** |  |  |  |  |  |
| Outcomes |  |  |  |  |  |  |  |
| Physical examination | **√** | **√** | **√** | **√** | **√** | **√** | **√** |
| Scale for gastrointestinal symptoms | **√** | **√** | **√** | **√** | **√** | **√** | **√** |
| Scale and evaluation for PD |  | **√** |  |  | **√** | **√** | **√** |
| Safety evalation |  |  |  |  |  |  |  |
| Routine blood test | **√** | **√** | **√** |  |  |  | **√** |
| Liver function | **√** | **√** | **√** |  |  |  | **√** |
| Adverse event |  | **√** | **√** | **√** | **√** | **√** | **√** |
| Other |  |  |  |  |  |  |  |
| Randomization |  | **√** |  |  |  |  |  |
| Donor selection |  | **√** | **√** | **√** |  |  |  |
| Microbiota analysis（16S rRNA and metagenomics sequencing） |  | **√** | **√** | **√** | **√** | **√** | **√** |

**Abstract**

| **Title:**  Fecal microbiota transplantation for researching the therapeutic effect of gut micro-ecology in Parkinson’s disease: a clinic study  基于粪菌移植（FMT）技术探索肠道微生态环境在帕金森病患者中疗效价值的临床研究 |
| --- |
| **Objectiv:**  In this study, fecal bacteria transplantation (FMT) was used to reconstruct the intestinal microecology for the treatment of Parkinson's disease, and combined with 16sRNA sequencing and metagenomic sequencing technology, the relevant mechanism of the effectiveness of intestinal microecology in the treatment of Parkinson's disease was studied. |
| **Participants:**  56 Patient with Parkinson’s disease (Hoehn-Yahr stage 1 to 3) |
| **Study design:**  This is a single center, placebo-controlled, randomized clinical trial.  Screened participants were randomly divided into two groups, in which the FMT group received fecal bacteria transplantation (FMT) through orally administrated capsules for consecutive 3 weeks, with 16 capsules at each time per week with an interval of 1 week, while the placebo group received the placebo capsule with the same protocol of FMT group, under dietary guide of physician. All participants were required to keep the original and usual medication, and follow-up at 4 weeks, 8 weeks and 12 weeks after the trial started. Specifically, the MDS-UPDRS score at weeks 0, 4, and 8, adverse effects at weeks 0, 4, 8, and 12, and evaluation for gastrointestinal disorders (including the IBS-SSS, GSRS, Bristol stool form scale, and IBS-QOL scale scores) and evaluation for mental health (including the PHQ-9 scale, GDS-15 scale, GAD-7 scale, Montreal Cognitive Assessment, Mini-mental State Examination scores) at weeks 0, 4 8 and 12 were recorded, and alterations in gut microbiota at weeks 0, 4 and 12 was analyzed by 16s rRNA and metagenomic sequencing. Meanwhile, all participants were treated routinely for concomitant diseases during treatment and follow-up. |
| **Criteria for inclusion and exclusion:**  **Inclusion criteria:**   1. Male and female aged 30 to 85 years; 2. Patients with early Parkinson's disease are definitely diagnosed as having h-y1 ~3 grade of Parkinson's disease in accordance with the diagnostic criteria for Parkinson's disease in China (2016 edition); 3. with normal communication skills; 4. Sign informed consent and agree to participate in this study.   **Exclusion criteria:**   1. Complicated with other serious diseases of the heart, liver, kidney, respiratory, digestive, blood and endocrine systems; 2. Combined with intestinal double infection, such as CDI, ehec, salmonella, shigella, campylobacter, plague, and cytomegalovirus; 3. Liver function was significantly abnormal or had the following liver disease history: AST or ALT was twice higher than the upper limit of normal value, history of liver cirrhosis, hepatic encephalopathy, history of esophageal varicose veins or portal shunt; 4. With evidence of renal damage or the following renal disease history: serum creatinine is 1.5 times higher than the upper limit of normal value; The history of dialysis; Or a history of nephrotic syndrome; 5. Patients with various acute infections, tumors and severe arrhythmias, mental disorders, drug or alcohol addiction; 6. Pregnant or lactating women; 7. Have used antibiotics or microecological agents in the past 4 weeks; 8. Patients with allergy or contraindications to "enterobacteria capsule" used in this study; 9. Clinical researchers with other related Parkinson's disease were being conducted at the time of enrollment or within 3 months before enrollment; 10. It is difficult to complete the interview, or various factors affecting the compliance. |
| **Intervention:**  FMT group (n=28) received 3 times of FMT treatment for consecutive 3 weeks, 16 FMT capsules were administrated orally each time, with an interval of 1 week. Placebo group (n=28) received 3 times of placebo treatment for consecutive 3 weeks, 16 placebo capsules were administrated orally each time, with an interval of 1 week. Patients in both groups were under dietary guide of the physician, and routinely treated with usual Parkinsonian medications and care. |
| **Outcomes:**  **Primary outcomes:**   - MDS-UPDRS score of the subjects at week 12.   **Secondary outcomes:**   - MDS-UPDRS score at weeks 0, 4, and 8. - Safety (adverse effects) at weeks 0, 4, 8, and 12. - Evaluation for gastrointestinal disorders (including the IBS-SSS, GSRS, Bristol stool form scale, and IBS-QOL scale scores) at weeks 0, 4, 8, and 12. - Evaluation for mental health (including the PHQ-9 scale, GDS-15 scale, GAD-7 scale, Montreal Cognitive Assessment, Mini-mental State Examination scores) at weeks 0, 4 8 and 12. - The change of gut microbiota after intervention at weeks 0, 4 and 12 by using 16s rRNA and metagenomic sequencing analysis. |
| **Bio-samples collection:**  **Stool collection:**  Stool from each participant before and after interventions were collect respectively, and stored at -80℃ for analyzing diversity change of fecal microbiota. |
|  |
| **Ethics:**  This trial protocol was permitted by IRB approval from Southwest Hospital affiliated with Army Medical University in Chongqing, China, all participants enrolled were signed with informed consent. All investigators strictly performed the protocol and accord with requirement from GCP. Legitimate rights and interest as well as safety of participants were guaranteed during the whole trial. |
| **Experimental cycle:**  Experimental cycle for each participant was 3 months totally, including wash out for 1 week, intervention for 3 weeks and follow up for 3 months. |

Trial Protocol

1. Background and Significance

Parkinson's disease (PD), a neurodegenerative disease commonly occurring in the elderly, is the second most common neurodegenerative disease, ranked only after Alzheimer's disease in its incidence. About 6 million people suffer from Parkinson's disease worldwide. The main clinical features of PD are motor disorders, including progressive motor retardation, muscular rigidity, quiescent tremor and abnormal postural gait, etc. In addition, PD can also be accompanied by a large number of non-motor symptoms (NMS), such as anosmia, depression, cognitive impairment, sleep disorder, nocturia, gastrointestinal dysfunction, etc.

However, the etiology and pathogenesis of Parkinson's disease are still not clear. At present, dopamine-based therapy is the most effective and commonly used treatment for PD, which can only partially alleviate the symptoms, and cannot block or delay the progression of the disease. Moreover, the abnormal symptoms and symptom fluctuations after long-term dopamine replacement therapy greatly affect the quality of life of patients.

Contrary to the traditional view, increasing data in recent years suggest that the pathological changes of PD do not originate from the brain, but probably originate from the gut, this consists to the imbalance of intestinal microflora of PD patients found in recent clinical studies. Recent studies have also shown that intestinal flora dysfunction may lead to α-syn misfolding, affecting all layers of neurological function including central nervous system, autonomic nervous system and intestinal peripheral nerves through the brain-gut axis, indicating a close link between non-motor symptoms and motor symptoms in PD patients. Although the exact role of intestinal flora in PD pathogenesis is unclear, but plenty of researches have proved that the occurrence of PD is associated with imbalance of intestinal flora. With increasing data showing that a bi-directional communication exists between gut and brain, the concept of "brain and intestine microbiota axis" has been proposed, the brain may affect gut by neural and endocrine signaling molecules, conversely, gut microbiota may in turn modulate neurological function through metabolites and cytokines. To explore the influence of microbiota dysbiosis on Parkinson's disease and study the effect of gut microbiota on PD patient might contribute to understand the pathogenesis and improve treatment of PD from a new perspective, and to provide new insights for its clinical prevention and management. More and more studies have focused on food therapy and probiotics therapy that are beneficial to the restoration of gut microbiota, as well as animal experimental studies of fecal microbiota transplantation (FMT) for PD.

Probiotics can be beneficial for the gastrointestinal motor function, mental cognition, anxiety, depression, and immune regulation of PD. Studies have shown that constipation and intestinal peristalsis are direct risk factors for PD [1], which can increase the incidence of PD by 2-4 times. As a microbial adjuvant therapy for PD, probiotics have been proved to be effective in gastrointestinal function, mental cognition, anxiety and depression, and immune regulation in PD patients. Preliminary studies have shown that administration of yoghurt containing *Lactobacillus caseus* [2] can improve stool characteristics and bowel frequency in PD patients. Relative studies of underlying mechanisms have shown that probiotics can directly stimulate intestinal smooth muscle cells and thus increase intestinal vitality [3]. About 24.3% of PD patients have gastrointestinal symptoms of irritable bowel syndrome (IBS) [4]. IBS like symptoms of PD patients are related to the decreased abundance of *Prevotella* which is verified by stool specimens of patients [4]. The degree of fecal drying and the severity of constipation are related to the diversity of gut microbiota [5], moreover, the use of probiotics reduces THE MS-UPDRS score in PD patients [6]. However, its effect on neurological physiology is still unknown. Dementia and cognitive impairment are common symptoms and complications of Parkinson's disease, among which cognitive dysfunction is manifested at the early stage of Parkinson's disease [6]. A large number of studies have confirmed that PD and AD (Alzheimer's disease) have overlapping pathogenesis and pathological manifestation. Studies on cognitive impairment have shown that *Bifidobacterium brevis* [8] could reversed the cognitive impairment in mice with Alzheimer's disease [9]. Probiotics Supplementation with mixed species products including *Lactobacillus acidophilus*, *Lactobacillus casei*, *Bifidobacterium fermentum* improved MMSE scores in Alzheimer's patients. In addition, depression and anxiety are also very common in PD patients [10], which are also the high incidence of prodromal symptoms in PD patients. Animal experiments have proved that *Lactobacillus plantarum* PS128 can up-regulate the serotonin level of dopamine in the striatum of sterile mice, and has a significant effect on improving their anxiety and depression [11]. There is also evidence showing that probiotics may be effective in the treatment of PD depression or anxiety, but it remains to be confirmed by conclusive scientific evidence. Additionally, a few studies have shown that probiotics can also improve Parkinson's disease through immunomodulation [12, 13]. However, many issues remained on the proper use of probiotics to improve PD, including which types of probiotics, the dose and duration of treatment, and the efficacy of probiotics colonization in the gut.

Fecal Microbiota transplantation (FMT) refers to transplantation of bacteria from feces of healthy donor into the gastrointestinal tract of recipient patients, for the purpose of reconstructing gut microbiota, and treatment of intestinal and extra-intestinal diseases. At present, a consensus exists that intestinal microecosystem is a special organ of human beings, and its abnormality is closely related to various diseases intra- and extra-intestine. Therefore, FMT is also called a special "organ transplantation", where gut microbiota is an organ that can be shared by human beings without immune rejection concerns.

Disruption of gut microbiota and changed fecal short-chain fatty acids (SCFAs) were observed in MPTP-treated PD mice model. FMT from healthy mice to MPTP-treated PD mice can inhibit glial cell activation and neuroinflammation, and play a protective role on nerves [14]. It is well known that SCFAs can aggravate exercise symptoms in PD mice, while its concentration in feces of FMT mice decreases. The long-term effects and potential adverse reactions of FMT on PD patients have not been clearly reported yet. FMT can rebuild intestinal flora, elevate intestinal microbial diversity, and restore the overall gut function. Compared with host genes, environmental factors such as diet have a greater impact on intestinal flora [15]. Various studies have shown that FMT treatment can relieve constipation symptoms in patients with slow transit constipation [16]. FMT combined with soluble dietary fiber can provide short-term and long-term treatment for slow transit constipation in the general population [17].

Our group have developed novel technology for "fecal bacteria transplant" firstly by using FMT capsule in southwest China, and is the only affiliation to conduct this technology. Through continuous accumulation of clinical practice, more than 2000 cases were successfully treated, including the overwhelming clostridium infection (pseudo membrane enteritis), where 112 cases of patients reached 100% efficient rate, our technology also conducted on inflammatory bowel disease among 516 patients with responsive rate of 75%, meanwhile, the effective rate was also 100% in 79 patients with small intestinal bacterial overgrowth, and 76% in 60 patients with autism, 78% in 681 patients with intractable irritable bowel syndrome (IBS), and 77% in 559 patients with intractable constipation, 74% in 67 patients with chronic fatigue syndrome, 73% in 58 patients with intestinal failure, 65% in 42 patients with metabolic syndrome. All the clinical effect is desirable with no serious adverse event occurred. In 2015, this new technology of FMT capsule was authorized as the New clinical Technology of the Third Military Medical University, class II and Class A new technology. In clinical practice, our research group independently created FMT capsule named as “FL-001” (see Figure 1), which has been authorized by the national invention patent in China (Patent No.: 2015103040414), has been successfully applied in clinical practice, the successful implementation of FMT was conducted among 2000 cases, where more than 1000 patients were performed through “FL - 001", others through the nasojejunal nutrient canal or other manner, with equivalent efficacy.


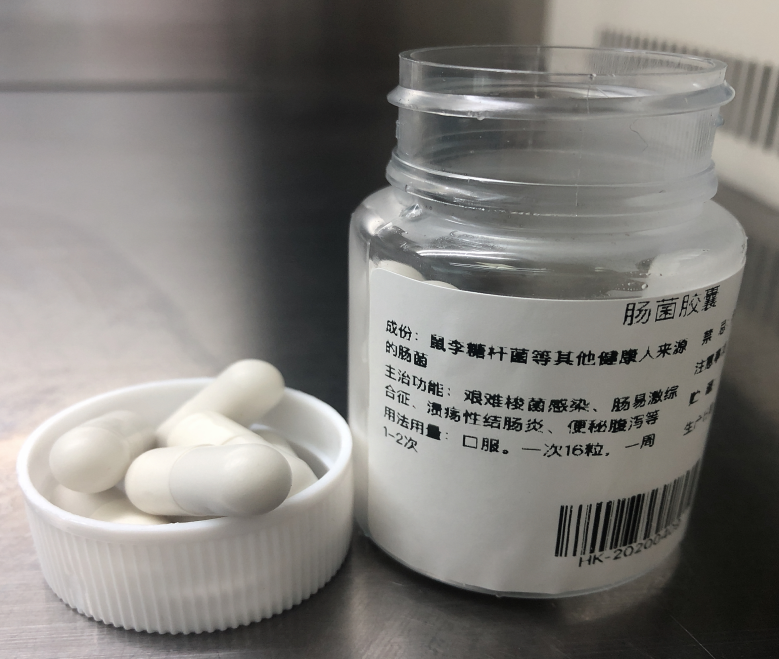


Figure 1. The photograph of FMT capsules “FL-001”

FMT capsule “FL-001" has carried out a series of standardized construction in the aspects of convenient acquisition of fecal bacteria, quantity of transplantation, easy process, effective reduction of bacterial death, etc., which has significantly improved the efficiency and convenience of fecal bacteria, expanded its application scope, and thus benefit for promotion and application of FMT. However, few studies have been reported on Parkinson's disease by FMT, according to available literature, no placebo-controlled, randomized clinical trial has been reported on FMT for the treatment of Parkinson's disease worldwide.

This trial intends to conduct a randomized controlled clinical study on Parkinson's disease patient with H-Y stage 1-3, focusing on the efficacy and safety of FMT capsule in the treatment of Parkinson's disease.

1. Objectives of Study

In this study, fecal bacteria transplantation (FMT) was used to reconstruct the intestinal microecology for the treatment of Parkinson's disease, and combined with 16sRNA sequencing and metagenomic sequencing technology, the relevant mechanism of the effectiveness of intestinal microecology in the treatment of Parkinson's disease was studied.

1. Study Design

This is a single center, placebo-controlled, randomized clinical trial.

Screened participants were randomly divided into two groups in a 1:1 ratio by using computer-generated random numbers, in which the FMT group received fecal bacteria transplantation (FMT) through orally administrated capsules for consecutive 3 weeks, with 16 capsules at each time per week with an interval of 1 week, while the placebo group received the placebo capsule with the same protocol of FMT group, under dietary guide of physician. All participants were required to keep the original and usual medication, and follow-up at 4 weeks, 8 weeks and 12 weeks after the trial started. Specifically, the MDS-UPDRS score at weeks 0, 4, and 8, adverse effects at weeks 0, 4, 8, and 12, and evaluation for gastrointestinal disorders (including the IBS-SSS, GSRS, Bristol stool form scale, and IBS-QOL scale scores) and evaluation for mental health (including the PHQ-9 scale, GDS-15 scale, GAD-7 scale, Montreal Cognitive Assessment, Mini-mental State Examination scores) at weeks 0, 4 8 and 12 were recorded, and alterations in gut microbiota at weeks 0, 4 and 12 was analyzed by 16s rRNA and metagenomic sequencing. Meanwhile, all participants were treated routinely for concomitant diseases during treatment and follow-up.

Principle of dose of FMT capsule: administration of FMT capsule is based on the number of living bacteria in the capsule. The dose of bacteria in 16 capsules equals to the dose of bacteria in the 50g stool. According to FMT guidelines and previous published clinical data, the amount of stool corresponding to each fecal bacteria transplantation should be controlled between 25g-50g [18-22]. At present, there is no standardized regulation and specification for consistent dose and course of FMT. According to the colonization of previous studies on FMT experiment, the recipient's microbiota shifts to the donor at one week after FMT [23]. Clinical studies on treatment of IBS by FMT showed that the effective rate was 85% ~ 70% in the short term (1-3 months) and 45% ~ 70% in the long term (6 months ~1 year) [24-27]. The respondents experienced considerable relief of symptoms and improved quality of life. This shows that the effect of FMT would wear off, indicating that FMT needs to be repeated regularly. Thus, in our trial, the dose was 16 capsules per week for 3 weeks, with interval of 1 week.

In order to minimize the interference of dietary and environmental factors on gut microbiota, participants included in the study were required to have regular and light diet, avoid spicy, greasy, raw, cold and hard food, and use of any probiotics and antibiotics is strictly prohibited.

All participants were treated routinely for concomitant diseases during treatment and follow-up.

1. Fecal Microbiota Transplantation (FMT)

Fecal Microbiota transplantation (FMT) is defined as transplantation of functional bacteria in feces of healthy donors into the gut of patients to restore gut microbiota and relieve intestinal and extra-intestinal disorders. Currently, the use of FMT can be divided into two types: routine treatment of medically recommended diseases (third recurrence of Clostridium difficile infection) and clinic studies of diseases beyond the guidelines (i.e., bacteria-related diseases). Till December 1, 2016, the Chinese official clinical trial registry www.clinicaltrials.gov had registered 140 clinical trials for FMT involving the treatment of CDI, IBD, irritable bowel syndrome, cirrhosis, fatty liver, hepatic encephalopathy, epilepsy, obesity, nutritional and metabolic abnormalities, organ transplantation and dozens of other diseases.

As an effective means for gut microbiota restoration, Fecal microbiota transplantation (FMT) has been used for the treatment and explorative research of a variety of gastrointestinal diseases such as *Clostridium diffcile* infection, and is regarded as a breakthrough medical progress in recent years. However, this is not a technology, but a system.

1. Participants:

A total of 56 patients diagnosed with early Parkinson’s disease were enrolled, in a 1:1 ratio of FMT group and placebo group in a 1:1 ratio by using computer-generated random numbers.

- 1. Sample size:

According to current reports, the therapeutic efficacy on PD patients is about 48%, and the estimated efficacy of FMT on PD patients is 80%. Assuming the allocation of 1:1, type 1 error of 0.05 (α), and power of 0.9 (1-β), based on former reports about conventional treatment and FMT on Parkinson's disease [28,29], 56 participants were enrolled assuming a 10% dropout rate to detect a minimum difference in MDS-UPDRS scores between the FMT and placebo groups.

- 1. Inclusion criteria:

1. Male and female aged 30 to 85 years;
2. Patients with early Parkinson's disease are definitely diagnosed as having h-y1 ~3 grade of Parkinson's disease in accordance with the diagnostic criteria for Parkinson's disease in China (2016 edition);
3. with normal communication skills;
4. Sign informed consent and agree to participate in this study.

5.2 Exclusion criteria:

1. Complicated with other serious diseases of the heart, liver, kidney, respiratory, digestive, blood and endocrine systems;
2. Combined with intestinal double infection, such as CDI, ehec, salmonella, shigella, campylobacter, plague, and cytomegalovirus;
3. Liver function was significantly abnormal or had the following liver disease history: AST or ALT was twice higher than the upper limit of normal value, history of liver cirrhosis, hepatic encephalopathy, history of esophageal varicose veins or portal shunt;
4. With evidence of renal damage or the following renal disease history: serum creatinine is 1.5 times higher than the upper limit of normal value; The history of dialysis; Or a history of nephrotic syndrome;
5. Patients with various acute infections, tumors and severe arrhythmias, mental disorders, drug or alcohol addiction;
6. Pregnant or lactating women;
7. Have used antibiotics or microecological agents in the past 4 weeks;
8. Patients with allergy or contraindications to "enterobacteria capsule" used in this study;
9. Clinical researchers with other related Parkinson's disease were being conducted at the time of enrollment or within 3 months before enrollment;
10. It is difficult to complete the interview, or various factors affecting the compliance.

5.3 Withdrawal criteria

In accordance with the GCP guidelines, all participants have the right to withdraw from the trial at any time, and will withdraw from the trial if any event of the following occurs during the trial.

1) Toxicity intolerance;

2) The participant is considered to be unsuitable to continue using the experimental intervention;

3) The participant withdraws informed consent;

4) Severe deviation or violation of the program occurs, and affect the drug safety evaluation.

6. Trial Procedure and Outcomes

- 1. Trial Procedure

6.1.1 Wash out ( -1~0 week)

Discontinue all active gastrointestinal motility drugs, laxatives, probiotics, and other related drugs.

1) Sign the informed consent.

2) Acquisition of medical history: medical history and treatment history, concomitant diseases and medications.

3) Physical examination: blood pressure, heart rate, temperature, respiratory rate, height, weight and scale for abdominal pain.

4) Laboratory examination 1: routine blood tests.

5) Laboratory examination 2: liver, renal function and blood homocysteine examination.

6) Participant screening according to inclusion/exclusion criteria based on examination results within one week.

7) Randomization of enrolled participants.

6.1.2 Baseline information (0 week)

Participants who have not received relevant drug or treatment within 4 weeks, or have received other relevant treatment within 4 weeks but already passed wash out period, meanwhile passed the screening based on clinical symptoms, basic laboratory examination and V0 examination results, and met the inclusion criteria were randomized into the random group for following baseline examination.

6.1.2.1 Scales for clinical symptoms

1. Gastrointestinal symptoms:

| Index | Rating | Score |  |
| --- | --- | --- | --- |
| Abdominal pain | No□ Yes□ area_____ |  | Table.1 |
| Change of stool manifestation | No□ Yes□___ time/day |  | Table.2 |
| IBS-SSS | Mild□ Moderate□ Severe□ |  | Table.3 |
| GSRS |  |  | Table.4 |

1. Quality of life evaluation:

| Index | Score |  |
| --- | --- | --- |
| IBS-QOL |  | Table.5 |
| QOL |  | Table.6 |

1. Evaluation for anxiety, depression and cognition:

| Index | Score |  |
| --- | --- | --- |
| PHQ-9 |  | Table.7 |
| GDS-15 |  | Table.8 |
| GAD-7 |  | Table.9 |
| MOCA |  | Table.10 |
| MMSE |  | Table.11 |

1. Evaluation for Parkinsonism

| Index | Score |  |
| --- | --- | --- |
| UPDRS-1 |  | Table.12 |
| UPDRS-2 |  | Table.13 |
| UPDRS-3 |  | Table.14 |
| UPDRS-4 |  | Table.15 |

6.1.2.2 Fecal microbiota analysis before interventions, by using 16s rDNA sequencing.

6.1.3 Interventions (1-3 week):

Participants were randomly assigned to the FMT group or the placebo group, FMT group received 3 times of FMT treatment for consecutive 3 weeks, 16 FMT capsules were administrated orally each time, with an interval of 1 week. Placebo group received 3 times of placebo treatment for consecutive 3 weeks, 16 placebo capsules were administrated orally each time, with an interval of 1 week. Patients in both groups were routinely treated with usual Parkinsonian medications and care.

During this period, participants in two groups were kept the original and conventional medication, and visited at 4 weeks, 8 weeks and 12 weeks respectively after the trial started, to observe the changes of clinical indicators and gut microbiota. Specifically, the MDS-UPDRS score at weeks 0, 4, and 8, adverse effects at weeks 0, 4, 8, and 12, and evaluation for gastrointestinal disorders (including the IBS-SSS, GSRS, Bristol stool form scale, and IBS-QOL scale scores) and evaluation for mental health (including the PHQ-9 scale, GDS-15 scale, GAD-7 scale, Montreal Cognitive Assessment, Mini-mental State Examination scores) at weeks 0, 4 8 and 12 were recorded, and alterations in gut microbiota at weeks 0, 4 and 12 was analyzed by 16s rRNA and metagenomic sequencing. Meanwhile, all participants were treated routinely for concomitant diseases during treatment and follow-up.

6.1.4 End point (3 month)

At the end of the trial, participants received a comprehensive examination to evaluate the efficacy and safety of FMT capsules in the treatment of Parkinson's disease. The change of gut microbiota and effective colonization duration of FMT on Parkinson's disease patients will be analyzed through 16s rDNA, metagenomic sequencing and bioinformatics analysis.

6.2 Follow up

Follow-up form for Parkinson disease

Time _______

| Basic information | Name: Gender: Age: | | |
| --- | --- | --- | --- |
|  | Address: Telephone: | | |
|  | Height: ____cm Body weight: ____kg BMI: ____kg/cm^2^  Blood pressure: ____mmHg Heart beat: ____times/min  Temperature: ____℃  Respiratory rate: ____times/min | | |
| Medical history | Time of onset: Time of diagnosis: | | |
|  | Diagnosis: | | |
| Medication history | Drug 1. Laxative:  Dosage: ____times/day ___mg/time Duration: _____ | | |
|  | Drug 1. Bowel dynamic regulator:  Dosage: ____times/day ___mg/time Duration: _____ | | |
|  | Drug 1. Probiotics:  Dosage: ____times/day ___mg/time Duration: _____ | | |
|  | Medical history: | | |
| Concomitant drug |  | | |
| Compliance of medication | 1.Regular 2. Discontinuous 3. No medication | | |
| Symptom regularity | 1.Repeated 2. Continuous 3. Disappeared | | |
| Personal habit | Smoke: No□ Yes□ ____ times/day | | |
|  | Alcohol: No□ Yes□ ____mL/day | | |
|  | Dietary: 1. Good 2. Common 3. Bad | | |
|  | Sleep: 1. Good 2. Common 3. Bad | | |
| Intervention | FMT group： Date______ Time______ | | |
|  | Placebo group： Date______ Time______ | | |
| Clinical symptoms | 1. **Gastrointestinal symptoms:**  \| Abdominal pain \| No□ Yes□ area____ \| Score: ___ \| Table.1 \| \| --- \| --- \| --- \| --- \| \| Change of stool manifestation \| No□ Yes□___ time/day \| Score: ___ \| Table.2 \| \| IBS-SSS \| Mild□ Moderate□ Severe□ \| Score: ___ \| Table.3 \| \| GSRS \|  \| Score: ___ \| Table.4 \|  1. **Quality of life：**  \| IBS-QOL \| Score: ___ \| Table.5 \| \| \| --- \| --- \| --- \| --- \| \| QOL \| Score: ___ \| Table.6 \|  1. **Anxiety, depression and cognition:**  \| PHQ-9 \| Score: ___ \| Table.7 \| \| --- \| --- \| --- \| \| GDS-15 \| Score: ___ \| Table.8 \| \| GAD-7 \| Score: ___ \| Table.9 \| \| MOCA \| Score: ___ \| Table.10 \| \| MMSE \| Score: ___ \| Table.11 \|  1. **Parkinsonism:**  \| UPDRS-1 \| Score: ___ \| Table.12 \| \| --- \| --- \| --- \| \| UPDRS-2 \| Score: ___ \| Table.13 \| \| UPDRS-3 \| Score: ___ \| Table.14 \| \| UPDRS-4 \| Score: ___ \| Table.15 \|   Others: | | |
|  |  | Investigator |  |

Laboratory Examinations before FMT

| **Routine blood test (Date: ___________)** | | | | | | |
| --- | --- | --- | --- | --- | --- | --- |
| WBC |  | ×10^9^/L | MONO |  | ×10^9^/L | |
| NEUT |  | % | MONO |  | % | |
| RBC |  | ×10^12^/L | PLT |  | ×10^9^/L | |
| Hb |  | g/L | CRP |  | mg/L | |
| **Serological examination (Date：____________)** | | | | | | |
| ALT |  | IU/L | GGT |  | | IU/L |
| AST |  | IU/L | Scr |  | | μmol/L |
| ALP |  | IU/L | BUN |  | | mmol/L |
| TBIL |  | μmol/L | UA |  | | μmol/L |
| Cys C |  | mg/L | Hcy |  | | μmol/L |
| Gut microbiota（16S rDNA） | | | | | | |
| Cerebral fMRI: | | | | | | |

Follow-up Form after FMT (week 4)

| Clinical symptoms | 1. **Gastrointestinal symptoms:**  \| Abdominal pain \| No□ Yes□ area____ \| Score: ___ \| Table.1 \| \| --- \| --- \| --- \| --- \| \| Change of stool manifestation \| No□ Yes□___ time/day \| Score: ___ \| Table.2 \| \| IBS-SSS \| Mild□ Moderate□ Severe□ \| Score: ___ \| Table.3 \| \| GSRS \|  \| Score: ___ \| Table.4 \|  1. **Quality of life：**  \| IBS-QOL \| Score: ___ \| Table.5 \| \| \| --- \| --- \| --- \| --- \| \| QOL \| Score: ___ \| Table.6 \|  1. **Anxiety, depression and cognition:**  \| PHQ-9 \| Score: ___ \| Table.7 \| \| --- \| --- \| --- \| \| GDS-15 \| Score: ___ \| Table.8 \| \| GAD-7 \| Score: ___ \| Table.9 \| \| MOCA \| Score: ___ \| Table.10 \| \| MMSE \| Score: ___ \| Table.11 \|  1. **Parkinsonism:**  \| UPDRS-1 \| Score: ___ \| Table.12 \| \| --- \| --- \| --- \| \| UPDRS-2 \| Score: ___ \| Table.13 \| \| UPDRS-3 \| Score: ___ \| Table.14 \| \| UPDRS-4 \| Score: ___ \| Table.15 \|   Others:  Change of feeling:  Current medication: | | |
| --- | --- | --- | --- | --- | --- | --- | --- | --- | --- | --- | --- | --- | --- | --- | --- | --- | --- | --- | --- | --- | --- | --- | --- | --- | --- | --- | --- | --- | --- | --- | --- | --- | --- | --- | --- | --- | --- | --- | --- | --- | --- | --- | --- | --- | --- | --- | --- | --- | --- | --- | --- | --- | --- |
|  | Gut microbiota | | |
| Adverse events |  | | |
|  |  | Investigator |  |

Follow-up Form after FMT (week 8)

| Clinical symptoms | 1. **Gastrointestinal symptoms:**  \| Abdominal pain \| No□ Yes□ area____ \| Score: ___ \| Table.1 \| \| --- \| --- \| --- \| --- \| \| Change of stool manifestation \| No□ Yes□___ time/day \| Score: ___ \| Table.2 \| \| IBS-SSS \| Mild□ Moderate□ Severe□ \| Score: ___ \| Table.3 \| \| GSRS \|  \| Score: ___ \| Table.4 \|  1. **Quality of life：**  \| IBS-QOL \| Score: ___ \| Table.5 \| \| \| --- \| --- \| --- \| --- \| \| QOL \| Score: ___ \| Table.6 \|  1. **Anxiety, depression and cognition:**  \| PHQ-9 \| Score: ___ \| Table.7 \| \| --- \| --- \| --- \| \| GDS-15 \| Score: ___ \| Table.8 \| \| GAD-7 \| Score: ___ \| Table.9 \| \| MOCA \| Score: ___ \| Table.10 \| \| MMSE \| Score: ___ \| Table.11 \|  1. **Parkinsonism:**  \| UPDRS-1 \| Score: ___ \| Table.12 \| \| --- \| --- \| --- \| \| UPDRS-2 \| Score: ___ \| Table.13 \| \| UPDRS-3 \| Score: ___ \| Table.14 \| \| UPDRS-4 \| Score: ___ \| Table.15 \|   Others:  Change of feeling:  Current medication: | | |
| --- | --- | --- | --- | --- | --- | --- | --- | --- | --- | --- | --- | --- | --- | --- | --- | --- | --- | --- | --- | --- | --- | --- | --- | --- | --- | --- | --- | --- | --- | --- | --- | --- | --- | --- | --- | --- | --- | --- | --- | --- | --- | --- | --- | --- | --- | --- | --- | --- | --- | --- | --- | --- | --- |
| Adverse events |  | | |
|  |  | Investigator |  |

Follow-up Form after FMT (week 12)

| Clinical symptoms | | 1. **Gastrointestinal symptoms:**  \| Abdominal pain \| No□ Yes□ area____ \| Score: ___ \| Table.1 \| \| --- \| --- \| --- \| --- \| \| Change of stool manifestation \| No□ Yes□___ time/day \| Score: ___ \| Table.2 \| \| IBS-SSS \| Mild□ Moderate□ Severe□ \| Score: ___ \| Table.3 \| \| GSRS \|  \| Score: ___ \| Table.4 \|  1. **Quality of life：**  \| IBS-QOL \| Score: ___ \| Table.5 \| \| \| --- \| --- \| --- \| --- \| \| QOL \| Score: ___ \| Table.6 \|  1. **Anxiety, depression and cognition:**  \| PHQ-9 \| Score: ___ \| Table.7 \| \| --- \| --- \| --- \| \| GDS-15 \| Score: ___ \| Table.8 \| \| GAD-7 \| Score: ___ \| Table.9 \| \| MOCA \| Score: ___ \| Table.10 \| \| MMSE \| Score: ___ \| Table.11 \|  1. **Parkinsonism:**  \| UPDRS-1 \| Score: ___ \| Table.12 \| \| --- \| --- \| --- \| \| UPDRS-2 \| Score: ___ \| Table.13 \| \| UPDRS-3 \| Score: ___ \| Table.14 \| \| UPDRS-4 \| Score: ___ \| Table.15 \|   Others: | | | | | | |
| --- | --- | --- | --- | --- | --- | --- | --- | --- | --- | --- | --- | --- | --- | --- | --- | --- | --- | --- | --- | --- | --- | --- | --- | --- | --- | --- | --- | --- | --- | --- | --- | --- | --- | --- | --- | --- | --- | --- | --- | --- | --- | --- | --- | --- | --- | --- | --- | --- | --- | --- | --- | --- | --- | --- | --- | --- | --- | --- |
| **Routine blood test (Date: ___________)** | | | | | | | | |
| WBC |  | | ×10^9^/L | MONO | |  | | ×10^9^/L |
| NEUT |  | | % | MONO | |  | | % |
| RBC |  | | ×10^12^/L | PLT | |  | | ×10^9^/L |
| Hb |  | | g/L | CRP | |  | | mg/L |
| **Serological examination (Date：____________)** | | | | | | | | |
| ALT |  | | IU/L | | GGT | |  | IU/L |
| AST |  | | IU/L | | Scr | |  | μmol/L |
| ALP |  | | IU/L | | BUN | |  | mmol/L |
| TBIL |  | | μmol/L | | UA | |  | μmol/L |
| Cys C |  | | mg/L | | Hcy | |  | μmol/L |
| Gut microbiota | | | | | | | | |
| Adverse events | |  | | | | | | |
|  | |  | | | | Investigator | |  |

7. Outcomes:

7.1 Primary outcomes:

- MDS-UPDRS score of the subjects at week 12.

7.2 Secondary outcomes:

- MDS-UPDRS score at weeks 0, 4, and 8.
- Safety (adverse effects) at weeks 0, 4, 8, and 12.
- Evaluation for gastrointestinal disorders (including the IBS-SSS, GSRS, Bristol stool form scale, and IBS-QOL scale scores) at weeks 0, 4, 8, and 12.
- Evaluation for mental health (including the PHQ-9 scale, GDS-15 scale, GAD-7 scale, Montreal Cognitive Assessment, Mini-mental State Examination scores) at weeks 0, 4 8 and 12.
- The change of gut microbiota after intervention at weeks 0, 4 and 12 by using 16s rRNA and metagenomic sequencing analysis.

8. Adverse events:

8.1 Adverse events:

An adverse event (AE) is any unexpected sign, symptom, worsening disease, or other medical condition that occurs after the use of an experimental drug, even if they are not related to the experimental drug. "Experimental drug" is defined as the intervention under test and placebo used for the trial.

A timely and complete AE report can help a drug registration applicant determine whether it is likely to be a drug-related event, thereby, to learn more about drug toxicity, find out the dose-related toxicity of the drug, revise the trial plan, comply with regulations to protect participants, researchers and drug registration applicants.

Since participants signing informed consent, to 28 days after the last intervention, all the adverse events during this period must be recorded in the original medical documents and case report on the AE page, whether subjects self-reported, researchers asked, or is found by physical examination, laboratory examination, etc., or whether it is considered by the researchers is related to the study drug. If known, the disease or disorder involved (i.e., diagnosis) should be recorded, and the term commonly used for adverse events refers to CTCAE 4.03. All adverse events should be observed until resolved, stable, return to baseline, or the event is figured out to be attributed to drugs or factors unrelated to the trial, or when more information is unavailable. If the participants drop out of the trial and no more information is available, the time of drop-out should also be recorded.

The investigator must also provide the results of laboratory tests that is considered to be clinically significant or significantly abnormal to the applicant for drug registration and record them on the AE page of the original medical document and case report form. Whether or not it is related to the experimental intervention, the applicant for drug registration must be reported, as must any abnormal laboratory results that require or prolong hospitalization, if related to the trial or are considered clinically significant. Such abnormalities must be reported and treated in a timely manner if events are considered SAE.

Any medical condition or disease that existed before the trial is considered as adverse event only if it worsens after trial started.

The severity of an adverse event is a qualitative assessment of the scope or intensity of the adverse event as determined by the investigator or reported by the subject. Severity does not reflect the clinical severity of the event, but only describes the degree or range of pain or occurrence, nor does it reflect the association with the experimental drug.

Classification and evaluation criteria of severity of adverse events:

Classification standard: CTCAE version 4.03

Grade 1: Mild; asymptomatic or mild symptoms; clinical or diagnostic observations only; intervention not indicated.

Grade 2: Moderate; minimal, local or noninvasive intervention indicated; limiting age-appropriate instrumental ADL*.

Grade 3 Severe or medically significant but not immediately life-threatening; hospitalization or prolongation of hospitalization indicated; disabling; limiting self care ADL**.

Grade 4 Life-threatening consequences; urgent intervention indicated.

Grade 5 Death related to AE.

Criteria for determining the correlation between adverse events and experimental drugs:

The investigator should assess the possible association between adverse events and the intervention, with reference to the following classification criteria. (Table 23)

1) Relevant: the reaction conforms to the reasonable time sequence after intervention, and the known reaction of the intervention; the reaction is improved after discontinuation, and repeated after re-administration of the intervention, which could not be explained by the original disease or combination of medications of participants.

2) Probable relevant: the reaction conforms to the reasonable time sequence after intervention, and the known reaction of the intervention. The improvement after discontinuation could not be explained by the original disease or combination of medications of participants.

3) Possible relevant: the reaction conforms to the reasonable time sequence after intervention, and the known reaction of the intervention. The clinical status or other treatment of participants may also produce this reaction.

4) Possible irrelevant: the reaction did not conform to the reasonable time sequence after intervention, and the known reaction of the intervention. The clinical status or other treatment of participants may also produce this reaction.

5) Irrelevant: the reaction did not conform to the reasonable time sequence after intervention, and the known reaction of the intervention. The clinical status or other treatment of participants may also produce this reaction, and the reaction may improve or stop with the improvement of clinic status or discontinuation of other treatment, and it would be repeated after re-administration of other treatment.

Table 23. Criteria for relevence between adverse events and the trial

|  | Relevant | Probably relevant | Possibly relevant | Possibly irrelevant | Irrelevant |
| --- | --- | --- | --- | --- | --- |
| Conform to the reasonable time sequence after intervention | ＋ | ＋ | ＋ | — | — |
| Conform to the known reaction of the intervention | ＋ | ＋ | ± | — | — |
| Improve or disappear after intervention discontinued | ＋ | ＋ | ± | ± | — |
| Repeat after intervention re-administrated | ＋ | ？ | ？ | ？ | — |
| Not conform to original disease or combined medications | ＋ | ＋ | ± | ± | — |

+: positive, -: negative, ±: difficult to confirm or deny, ?: unknown.

8.2 Serious Adverse Events (SAE):

A serious adverse event (SAE) is defined as an adverse event that results in:

1) Death;

2) Life threatening;

3) Hospitalization or extension of hospitalization;

4) Persistent or severe disability or insufficiency;

5) Medical events that considered to be serious adverse events by investigators.

Serious Adverse Event refers to any untoward medical occurrence that at any dose:

1. results in death
2. is life-threatening
3. requires inpatient hospitalization or prolongation of existing hospitalization,
4. results in persistent or significant disability/incapacity, or
5. is considered serious by the investigator for a reason other than those listed.

If a serious adverse event occurs to the participants during the trial, whether or not it is related to the trial, investigators shall immediately take appropriate treatment to ensure participants’ safety. Investigators shall report the event immediately to the sponsor, the Ethics Committee, and the Food and Drug Administration within 24 hours. Meanwhile, researchers must report a serious adverse event form, and the time, severity, duration, measures taken and outcome of the serious adverse event should be included.

Serious adverse events that are not resolved at the end of the trial or drop out of participants must be followed up until one of the following conditions of SAE is achieved:

1) Resolved;

2) Stable;

3) Returns to baseline level (if a baseline value is available);

4) The event may be attributed to medication other than the trial or to factors unrelated to the trial, or when more information is unlikely to be available (the patient or healthcare provider refuses to provide additional information, or there the patient is still lost after best efforts have been made).

9. Sample Collection

Collection of stools

Stool of donors and participants before and after intervention were collected. 5g stool of each participants (the middle part which is not in contact with the air and the toilet after defecation) was collected with a stool collection tube, stored at -80℃, for assessing the change of microbiota before and after transplantation.

10. Participant management

10.1 Drop out

10.1.1 Criteria for drop out

In accordance with the GCP guidelines, all participants have the right to withdraw from the trial at any time, and participants will withdraw from the trial if any of the following occurs during the study period:

1. Toxicity intolerance;
2. The investigator considers that participant is not suitable to continue the trial;
3. Participant withdraws informed consent;
4. Severe deviation or violation of trial protocol occurs, and affects the safety evaluation for intervention.

10.2 Concomitant medication or treatment

10.2.1 Prohibited medication or treatment

During the whole trial, participants are required to not change their medication or treatment regimen without the investigator's guidance.

10.2.2 Allowed medication or treatment

The following situation may be considered as appropriate during the trial:

1. Concomitant diseases (such as hypertension and hyperlipidemia) require long-term medication;
2. Symptomatic treatment or supportive medication is required to control the symptoms of the disease in the event of adverse reactions due to treatment. All combined medication should be recorded in detail in the accompanying medication page of the case report form (CRF), indicating the reason, dose, and duration of medication.

11. Data management and statistical analysis

11.1 Data management

Case Report Form (CRF) is for recording clinical data in a clinical trial. All relevant data of each participant in the study should be timely and truthfully recorded by the investigator, and confirmed and signed. CRF is not allowed to change, and the investigator should sign and record the date of intervention change when necessary (see instructions for filling in). At the end of the trial, the CRF was retained by the clinical trial facility and sponsor of the investigator's hospital. After the completed CRF is reviewed by the clinical supervisor, the data will be entered and the content will not be modified. To protect the privacy of participants, name on the CRF shall be coded.

12. Preservation of data

Researchers should keep the data intact. All original data should be retained by the investigator until 5 years after the end of the trial.

13. Ethics

13.1 Ethics Committee

The rights, safety and health of the participants must take precedence over considerations of scientific and social interests. Ethics committee and informed consent are the main measures to protect the rights and interests of the participants.

The trial protocol shall be approved by the Ethics Committee before implemented. Any modification of the protocol during the trial shall be approved by the Ethics Committee. If serious adverse event occurs during the trial, it should be reported to the Ethics Committee promptly.

13.2 Informed consent

In informed consent, the investigator must explain the purpose, procedures, duration, potential risks and benefits, and any discomfort that may arise. Each participant must know that participation in the study is voluntary and that he/she may withdraw from the trial and withdraw his/her informed consent at any time without prejudice to his/her subsequent treatment or relationship with the physician.

Informed consent should be provided with standard written form and in non-professional language. Each informed consent must include all of the above content and include a voluntary statement. Informed consent shall be submitted to the ethics committee for approval.

After explaining the trial and confirming that each participant is well acknowledge of purpose of the trial, participants shall sign informed consent with date, and should read and consider their statements prior to signing. Participants will not be included in the trial without signed informed consent.

14. Responsibilities and Regulations

14.1 Investigators

The Investigator shall conscientiously perform the duties of the Investigator in accordance with all applicable regulations such as the GCP.

14.1.1 The investigator or designated representative must provide detailed information about the trial

1. Participants shall join the trial voluntarily and have the right to withdraw from the trial at any time without discrimination or retaliation, and their medical treatment, rights and interests will not be affected;
2. Participants must understand that their personal data during the trial will be kept confidential. If necessary, the Ethics Committee or the sponsor may have access to the data;
3. Purpose of the trial, process and duration of the trial, examination operation, possible benefits and risks shall be explained to participants;
4. Participants must be given sufficient time to consider whether they would like to participate in the trial. For participants who are unable to give consent, the above introduction and explanation shall be provided to their legal representative. The informed consent shall be given in the manner that the subject or legal representative can understand. During the trial, the participants shall have access to the relevant information at any time.
5. In case of trial related damage, participants will receive treatment and corresponding compensation.

14.1.2 Informed consent should be obtained after full and detailed explanations

1. The informed consent shall be signed and dated by the participant or his legal representative, and the investigator shall also sign the name and date on the informed consent;
2. For incapacitated participants, if the Ethics Committee agrees and the researcher considers that it is in the interest of the subjects to participate in the study, these patients can also be enrolled in the trial, and they should obtain the consent of their legal guardian, with signature and date.

14.1.3 Protection of participant’s privacy

This investigator will only collect and analyze data from subjects limited to those essential to the trial for relative efficacy, safety, quality, and application.

The collection and use of such data will fully ensure its confidentiality and comply with relevant laws and regulations to protect the participant's privacy.

The investigator shall ensure:

1. The data acquisition procedure is fair and legal;
2. The purpose of data acquisition is specific, and legal, and the data shall not be used in ways inconsistent with the purpose or otherwise contrary to the purpose of the trial;
3. The data acquisition is necessary and relevant to the research purpose, irrelevant data shall not be acquired;
4. The data acquisition shall be accurate and timely as necessary.
5. The investigator should be permitted by the participant before data acquisition.

Participants have the right to obtain personal data through the investigator and may request correction of errors or incomplete data. Appropriate responses from investigators to such requests shall be given, taking into account the purpose, the relevant trial procedure and relevant laws and regulations.

Appropriate regulatory procedure and measurement must be taken to protect the participant's personal information from unauthorized access and disclosure, accidental and illegal damage, and accidental loss and alteration. During the trial, investigator who have access to participant’s information and data shall keep confidential about them.

14.1.4 Protection of participant’s safety

The investigator is responsible for taking medical treatment related to the clinical trial and ensuring that participants receive appropriate medication in the event of adverse events.

It is the investigator's obligation to take all necessary treatments to ensure the participant's safety and to record relative information.

14.1.5 Guarantee of trial quality

The investigator must carefully read and understand the contents of the protocol in detail and strictly accord the protocol.

The investigator should ensure that sufficient time is available to conduct the trial within the time limit. The investigator shall explain the data, requirements, and responsibilities of the trial to all participated personnel, and ensure that sufficient participants are enrolled in accordance with the protocol.

The investigator shall ensure that the data are accurately, completely, timely and legally included in the medical records and case reports.

14.2 Cooperator

The cooperator shall, in accordance with all applicable regulations, conscientiously perform the duties and be responsible for financing and supervising the trial and, in particular, provide the investigator with legal guarantee for the financial compensation for the treatment of the participant who suffers any trial-related damage or death.

The sponsor shall file all the required documents in accordance with the regulations and shall be subject to the supervision of the regulatory agents.

14.3 Supervisor

The supervisor should accord with standard operating procedures (Sops) and supervise the clinical trial to ensure it is conducted in accordance with the protocol.

1. Prior to the trial, supervisor shall confirm that the performing unit has the appropriate qualification, including personnel allocation and training, equipped and well-functioning laboratory and relative conditions. Ensure that sufficient participants will participate in the trial and researchers will be accord with the requirements in the trial protocol;
2. Monitor the researchers' implementation of the protocol during the trial, confirm that all participants' informed consent has been obtained before enrollment, and the enrollment rate of the trial and the procedure of the trial, and confirm that the enrolled participants are accord with the participating criteria;
3. Confirm that all data records and reports are accurate and complete, and all case report forms are consistent with the original data.
4. Confirm that all adverse events are recorded and serious adverse events are reported and recorded within the required time;
5. Verify the supply, storage, distribution and reclamation of experimental drugs in accordance with relevant laws and regulations, and make corresponding records;
6. To assist the researcher in the necessary notification and application issues, and to report the data and results to the sponsor;
7. The follow-up visits, tests and examinations not performed by the researcher shall be clearly and accurately recorded, as well as whether errors or omissions are corrected;

14.4 Protocol modification

The protocol cannot be changed without the consent of the investigator and the cooperator. If the protocol must be modified, the modification or new version of the protocol (amendment) must be submitted to the Ethics Committee for approval prior to implementation, and submitted for approval by the local drug administration in accordance with the applicable requirements.

If there is an administrative amendment, the change must be submitted to the Ethics Committee or obtain official approval with writing version. If the protocol modification requires a change in the informed consent, the Ethics Committee must be notified. The application of the revised informed consent must be approved by the Ethics Committee.

14.5 Usage of information related to the trial

All unpublished data provided to the investigator by the cooperator, including but not limited to the operation, business information (such as patent situation, team, production process, basic research data, previous clinical data and prescription data, etc.) and any data generated from this trial shall be kept confidential.

The investigator shall keep this information confidential and to use it only for the purpose of performing this trial and not for any other purpose without the written consent of the cooperator.

Reference

[1] Gao X, Chen H, Schwarzschild M A, et al. A prospective study of bowel movement frequency and risk of Parkinson's disease[J]. Am J Epidemiol,2011,174(5):546-551.

[2] Cassani E, Privitera G, Pezzoli G, et al. Use of probiotics for the treatment of constipation in Parkinson's disease patients[J]. Minerva Gastroenterol Dietol,2011,57(2):117-121.

[3] Bar F, Von Koschitzky H, Roblick U, et al. Cell-free supernatants of Escherichia coli Nissle 1917 modulate human colonic motility: evidence from an in vitro organ bath study[J]. Neurogastroenterol Motil,2009,21(5):559-566, e16-e17.

[4] Mertsalmi T H, Aho V, Pereira P, et al. More than constipation - bowel symptoms in Parkinson's disease and their connection to gut microbiota[J]. Eur J Neurol,2017,24(11):1375-1383.

[5] Vandeputte D, Falony G, Vieira-Silva S, et al. Stool consistency is strongly associated with gut microbiota richness and composition, enterotypes and bacterial growth rates[J]. Gut,2016,65(1):57-62.

[6] Tamtaji O R, Taghizadeh M, Daneshvar K R, et al. Clinical and metabolic response to probiotic administration in people with Parkinson's disease: A randomized, double-blind, placebo-controlled trial[J]. Clin Nutr,2018.

[7] Pfeiffer H C, Lokkegaard A, Zoetmulder M, et al. Cognitive impairment in early-stage non-demented Parkinson's disease patients[J]. Acta Neurol Scand,2014,129(5):307-318.

[8] Akbari E, Asemi Z, Daneshvar K R, et al. Effect of Probiotic Supplementation on Cognitive Function and Metabolic Status in Alzheimer's Disease: A Randomized, Double-Blind and Controlled Trial[J]. Front Aging Neurosci,2016,8:256.

[9] Kobayashi Y, Sugahara H, Shimada K, et al. Therapeutic potential of Bifidobacterium breve strain A1 for preventing cognitive impairment in Alzheimer's disease[J]. Sci Rep,2017,7(1):13510.

[10] Aarsland D, Bronnick K, Ehrt U, et al. Neuropsychiatric symptoms in patients with Parkinson's disease and dementia: frequency, profile and associated care giver stress[J]. J Neurol Neurosurg Psychiatry,2007,78(1):36-42.

[11] Liu W H, Chuang H L, Huang Y T, et al. Alteration of behavior and monoamine levels attributable to Lactobacillus plantarum PS128 in germ-free mice[J]. Behav Brain Res,2016,298(Pt B):202-209.

[12] Brochard V, Combadiere B, Prigent A, et al. Infiltration of CD4+ lymphocytes into the brain contributes to neurodegeneration in a mouse model of Parkinson disease[J]. J Clin Invest,2009,119(1):182-192.

[13] Harms A S, Thome A D, Yan Z, et al. Peripheral monocyte entry is required for alpha-Synuclein induced inflammation and Neurodegeneration in a model of Parkinson disease[J]. Exp Neurol,2018,300:179-187.

[14] Sun M F, Zhu Y L, Zhou Z L, et al. Neuroprotective effects of fecal microbiota transplantation on MPTP-induced Parkinson's disease mice: Gut microbiota, glial reaction and TLR4/TNF-alpha signaling pathway[J]. Brain Behav Immun,2018,70:48-60.

[15] Rothschild D, Weissbrod O, Barkan E, et al. Environment dominates over host genetics in shaping human gut microbiota[J]. Nature,2018,555(7695):210-215.

[16] Tian H, Ge X, Nie Y, et al. Fecal microbiota transplantation in patients with slow-transit constipation: A randomized, clinical trial[J]. PLoS One,2017,12(2):e171308.

[17] Ge X, Tian H, Ding C, et al. Fecal Microbiota Transplantation in Combination with Soluble Dietary Fiber for Treatment of Slow Transit Constipation: A Pilot Study[J]. Arch Med Res,2016,47(3):236-242.

[18] Tian H, Ding C, Gong J, et al. Treatment of Slow Transit Constipation With Fecal Microbiota Transplantation: A Pilot Study[J]. J Clin Gastroenterol,2016,50(10):865-870.

[19] Ge X, Tian H, Ding C, et al. Fecal Microbiota Transplantation in Combination with Soluble Dietary Fiber for Treatment of Slow Transit Constipation: A Pilot Study[J]. Arch Med Res,2016,47(3):236-242.

[20] Kurokawa S, Kishimoto T, Mizuno S, et al. The effect of fecal microbiota transplantation on psychiatric symptoms among patients with irritable bowel syndrome, functional diarrhea and functional constipation: An open-label observational study[J]. J Affect Disord,2018,235:506-512.

[21] Mizuno S, Masaoka T, Naganuma M, et al. Bifidobacterium-Rich Fecal Donor May Be a Positive Predictor for Successful Fecal Microbiota Transplantation in Patients with Irritable Bowel Syndrome[J]. Digestion,2017,96(1):29-38.

[22] Ge X, Tian H, Ding C, et al. Fecal Microbiota Transplantation in Combination with Soluble Dietary Fiber for Treatment of Slow Transit Constipation: A Pilot Study[J]. Arch Med Res,2016,47(3):236-242.

[23] Mazzawi T, Lied G A, Sangnes D A, et al. The kinetics of gut microbial community composition in patients with irritable bowel syndrome following fecal microbiota transplantation[J]. PLoS One,2018,13(11):e194904.

[24] Borody T, Fischer M, Mitchell S, et al. Fecal microbiota transplantation in gastrointestinal disease: 2015 update and the road ahead[J]. Expert Rev Gastroenterol Hepatol,2015,9(11):1379-1391.

[25] Holvoet T, Joossens M, Wang J, et al. Assessment of faecal microbial transfer in irritable bowel syndrome with severe bloating[J]. Gut,2017,66(5):980-982.

[26] Pinn D M, Aroniadis O C, Brandt L J. Is fecal microbiota transplantation the answer for irritable bowel syndrome? A single-center experience[J]. Am J Gastroenterol,2014,109(11):1831-1832.

[27] Mazzawi T, Lied G A, Sangnes D A, et al. The kinetics of gut microbial community composition in patients with irritable bowel syndrome following fecal microbiota transplantation[J]. PLoS One,2018,13(11):e194904.

[28] van Kessel, S. P.; El Aidy, S., Contributions of Gut Bacteria and Diet to Drug Pharmacokinetics in the Treatment of Parkinson's Disease. Front Neurol 2019, 10, 1087.

[29] Warren, N.; O'Gorman, C.; Lehn, A.; Siskind, D., Dopamine dysregulation syndrome in Parkinson's disease: a systematic review of published cases. J Neurol Neurosurg Psychiatry 2017, 88 (12), 1060-1064.)
