# Supplementary material for: Efficacy of fecal microbiota transplantation in patients with Parkinson’s disease: clinical trial results from a randomized, placebo-controlled design
Source: Gut Microbes. 2023 Dec 6;15(2):2284247. doi: 10.1080/19490976.2023.2284247 (PMC10841011; doi:10.1080/19490976.2023.2284247)
Supplement: Supplemental Material [file KGMI_A_2284247_SM4073.zip › Supplementary tables (1).docx]

**Supporting table 1. Significantly different gut microbiota at phylum level between donor and PD patients (sorted in ascending order of p value).**

| **Phylum** | **Welch's t P-value** |
| --- | --- |
| p_Firmicutes | 3.75×10^4^ |
| p_Euryarchaeota | 1.48×10^3^ |
| p_Lentisphaerae | 1.36×10^2^ |
| p_Tenericutes | 4.32×10^2^ |

**Supporting table 2. Significantly different gut microbiota at genus level between donor and PD patients (sorted in ascending order of p value).**

| **Genus** | **Welch's t P-value** |
| --- | --- |
| g__Blautia | 1.23×10^7^ |
| g__Lachnospiraceae_FCS020_group | 8.83×10^7^ |
| g__Fusicatenibacter | 2.32×10^6^ |
| g__Erysipelotrichaceae_UCG-003 | 4.51×10^6^ |
| g__Enterococcus | 6.37×10^5^ |
| g__[Eubacterium]_hallii_group | 7.23×10^5^ |
| g__[Ruminococcus]_gnavus_group | 1.03×10^4^ |
| g__Butyricicoccus | 1.19×10^4^ |
| g__Lachnoclostridium | 1.23×10^4^ |
| g__Sutterella | 2.55×10^4^ |
| g__Anaerostipes | 2.70×10^4^ |
| g__[Eubacterium]_coprostanoligenes_group | 2.73×10^4^ |
| g__Eisenbergiella | 2.94×10^4^ |
| g__Alistipes | 2.99×10^4^ |
| g__Negativibacillus | 5.03×10^4^ |
| g__Lachnospiraceae_ND3007_group | 5.40×10^4^ |
| g__Veillonella | 8.15×10^4^ |
| g__Pseudomonas | 8.44×10^4^ |
| g__Agathobacter | 8.60×10^4^ |
| g__Anaerotruncus | 9.37×10^4^ |
| g__Dorea | 1.17×10^3^ |
| g__Methanobrevibacter | 1.31×10^3^ |
| g__uncultured | 1.42×10^3^ |
| g__Coprococcus | 1.48×10^3^ |
| g__Hafnia-Obesumbacterium | 1.56×10^3^ |
| g__Ochrobactrum | 3.14×10^3^ |
| g__Megamonas | 3.15×10^3^ |
| g__Alcaligenes | 3.24×10^3^ |
| g__UCG-002 | 3.68×10^3^ |
| g__[Eubacterium]_xylanophilum_group | 3.89×10^3^ |
| g__Oscillibacter | 5.18×10^3^ |
| g__Family_XIII_AD3011_group | 6.04×10^3^ |
| g__Odoribacter | 7.23×10^3^ |
| g__Brevundimonas | 7.24×10^3^ |
| g__Acidaminococcus | 7.36×10^3^ |
| g__Butyricimonas | 7.56×10^3^ |
| g__UBA1819 | 8.36×10^3^ |
| g__Bacillus | 9.38×10^3^ |
| g__Streptococcus | 1.11×10^2^ |
| g__UCG-005 | 1.13×10^2^ |
| g__Defluviitaleaceae_UCG-011 | 1.44×10^2^ |
| g__Dubosiella | 1.86×10^2^ |
| g__Hungatella | 1.86×10^2^ |
| g__Colidextribacter | 2.02×10^2^ |
| g__Eubacterium | 2.06×10^2^ |
| g__Allisonella | 2.08×10^2^ |
| g__Lachnospiraceae_UCG-010 | 2.09×10^2^ |
| g__[Eubacterium]_ruminantium_group | 2.10×10^2^ |
| g__Christensenellaceae_R-7_group | 2.18×10^2^ |
| g__Acinetobacter | 2.50×10^2^ |
| g__Family_XIII_UCG-001 | 2.54×10^2^ |
| g__RF39 | 2.68×10^2^ |
| g__Desulfovibrio | 2.83×10^2^ |
| g__Achromobacter | 2.87×10^2^ |
| g__Erysipelatoclostridium | 2.90×10^2^ |
| g__Peptococcus | 3.25×10^2^ |
| g__Gemella | 3.91×10^2^ |
| g__Ruminococcus | 4.07×10^2^ |
| g__Clostridia_UCG-014 | 4.11×10^2^ |
| g__[Eubacterium]_siraeum_group | 4.81×10^2^ |
| g__NK4A214_group | 4.99×10^2^ |

**Supporting table 3**· Adverse effects occurred in the FMT responders and non-responders.

| **Adverse effects** | **FMT.R** | **FMT.NR** |
| --- | --- | --- |
| All events (n) | 2 | 1 |
| Stomach ache (n) | 0 | 0 |
| Nausea (n) | 1 | 0 |
| Headache (n) | 0 | 0 |
| Fatigue (n) | 0 | 0 |
| Dizziness (n) | 0 | 0 |
| Bloating (n) | 0 | 0 |
| Flatulence (n) | 1 | 0 |
| Diarrhea (n) | 0 | 1 |
| Mucus in the stool (n) | 0 | 0 |
| Obstipation (n) | 0 | 0 |
| Reﬂux (n) | 0 | 0 |
| Rash (n) | 0 | 0 |
| Vomiting (n) | 0 | 0 |
| Fever sensation (n) | 0 | 0 |
| Chest pain (n) | 0 | 0 |
| Fever (n) | 0 | 0 |
| Inﬂuenza symptoms (n) | 0 | 0 |
| Uncomfortable (n) | 0 | 0 |

**Supporting table 4· Clinic outcomes of FMT responders and non-responders.**

|  |  | **Score** | | **Group effect^d^** | | **Time effect^e^** | | **Group****×time effect^f^** | |
| --- | --- | --- | --- | --- | --- | --- | --- | --- | --- |
| Outcomes |  | FMT.R | FMT.NR | B (95% CI) | P | B (95% CI) | P | B (95% CI) | P |
| MDS-UPDRS^a^ | Week0 | 55.67 (15.56) | 47.87 (17.68) | 7.8 (-4.27, 19.87) | 0.21 | NA | NA | NA | NA |
|  | Week4 | 42.08 (14.12) | 44.33 (18.59) |  |  | -3.53 (-8.31, 1.25) | 0.15 | -10.05 (-17.40, -2.70) | * |
|  | Week8 | 42.17 (16.07) | 43.40 （16.37） |  |  | -4.47 (-0.15, 0.21) | 0.06 | -9.03 (-16.71, -1.35) | * |
|  | Week12 | 38.33 (18.93) | 44.80 （20.01） |  |  | -3.07 (-7.44, 1.30) | 0.17 | -14.27 (-22.83, -5.70) | ** |
| MDS-UPDRS 1^a^ | Week0 | 11.58 (4.94) | 10.73 (4.10) | 0.85 (-2.49, 4.19) | 0.62 | NA | NA | NA | NA |
|  | Week4 | 8.92 (3.45) | 7.87 (3.16) |  |  | -2.87 (-4.81, -0.92) | ** | 0.20 (-2.83, 3.23) | 0.9 |
|  | Week8 | 8.75 (5.56) | 8.33 （3.44） |  |  | -2.40 (-3.84, -0.92) | ** | -0.43 (-3.90, 3.03) | 0.81 |
|  | Week12 | 8.50 (6.61) | 8.27 （3.63） |  |  | -2.47 (-3.68, -1.26) | *** | -0.62 (-4.32, 3.09) | 0.74 |
| MDS-UPDRS 2^a^ | Week0 | 12.92 (4.46) | 11.33 (5.07) | 1.58 (-1.88, 5.04) | 0.37 | NA | NA | NA | NA |
|  | Week4 | 10.75 (4.27) | 11.27 （5.76） |  |  | -0.07 (-1.42, 1.28) | 0.92 | -2.10 (-4.62, 0.42) | 0.1 |
|  | Week8 | 10.17 (4.11) | 11.07 （6.15） |  |  | -0.27 (-1.61, 1.07) | 0.70 | -2.48 (-4.45, -0.51) | * |
|  | Week12 | 8.50 (4.44) | 11.27 （6.11） |  |  | -0.07 (-1.28, 1.15) | 0.91 | -4.35 (-5.76, -2.94) | *** |
| MDS-UPDRS 3^a^ | Week0 | 26.25 (10.41) | 23.53 (10.92) | 2.72 (-5.05, 10.48) | 0.49 | NA | NA | NA | NA |
|  | Week4 | 19.42 (9.17) | 22.93 （15.30） |  |  | -0.60 (-5.72, 4.52) | 0.82 | -6.23 (-12.32, -015) | * |
|  | Week8 | 19.67 (8.05) | 21.47 （10.94） |  |  | -2.07 (-6.13, 1.99) | 0.32 | -4.52 (-9.81, 0.78) | 0.09 |
|  | Week12 | 18.75 (10.23) | 22.60 （13.10） |  |  | -0.93 (-4.34, 2.47) | 0.59 | -6.57 (-12.18, -0.96) | * |
| MDS-UPDRS 4^a^ | Week0 | 4.92 (3.55) | 2.27 (2.55) | 2.65 (0.36, 4.94) | * | NA | NA | NA | NA |
|  | Week4 | 3.00 (2.56) | 2.27 （2.25） |  |  | 0.00 (-1.01, 1.01) | 1.00 | -1.92 (-3.48, -0.36) | * |
|  | Week8 | 3.58 (2.43) | 2.53 （2.23） |  |  | 0.27 (-0.74, 1.27) | 0.60 | -1.60 (-3.25, 0.05) | 0.06 |
|  | Week12 | 2.58 (2.50) | 2.67 （2.29） |  |  | 0.40 (-0.77, 1.57) | 0.50 | -2.73 (-4.43, -1.04) | ** |
| IBS-SSS^a^ | Week0 | 152.67 (92.19) | 117.93 (49.68) | 34.73 (-20.80, 90.27) | 0.22 | NA | NA | NA | NA |
|  | Week4 | 75.83 (83.72) | 38.67 (43.40) |  |  | -79.27 (-105.15, -53.38) | *** | 2.43 (-34.33, 39.20) | 0.90 |
|  | Week8 | 91.67 (86.95) | 56.53 (55.76) |  |  | -61.40 (-94.96, -27.84) | *** | 0.40 (-50.69, 51.49) | 0.99 |
|  | Week12 | 79.17 (63.88) | 53.20 (55.75) |  |  | -64.73 (-99.34, -20.13) | *** | -8.77 (-61.67, 44.14) | 0.75 |
| GSRS^a^ | Week0 | 8.83 (3.10) | 7.13 (2.56) | 1.70 (-0.39, 3.79) | 0.11 | NA | NA | NA | NA |
|  | Week4 | 4.88 (3.87) | 3.33 (3.00) |  |  | -3.80 (-4.99, -2.61) | *** | -0.16 (-2.02, 1.70) | 0.87 |
|  | Week8 | 3.96 (2.88) | 3.77 (3.02) |  |  | -3.37 (-4.61, -2.13) | *** | -1.51 (-3.65, 0.63) | 0.17 |
|  | Week12 | 3.67 (3.31) | 4.07 (3.03) |  |  | -3.07 (-4.49, -1.64) | *** | -2.10 (-3.89, -0.31) | * |
| IBS-QOL^a^ | Week0 | 27.25 (13.22) | 17.73 (15.06) | 9.52 (-0.75, 19.79) | 0.07 | NA | NA | NA | NA |
|  | Week4 | 11.25 (9.93) | 6.67 (7.51) |  |  | -11.07 (-18.31, -3.83) | ** | -4.93 (-13.20, 3.33) | 0.24 |
|  | Week8 | 9.25 (7.78) | 7.47 (7.82) |  |  | -10.27 (-17.35, 3.18) | ** | -7.73 (-17.24, 1.78) | 0.11 |
|  | Week12 | 10.00 (9.58) | 7.27 (8.45) |  |  | -10.47 (-17.87, -3.06) | ** | -6.78 (-15.69, 2.13) | 0.14 |
| Bristol score^b,c^ | Week0 | 1.5 (1, 3.75) | 2 (1, 4) | -0.34 (-1.99, 1.31) | 0.69 | NA | NA | NA | NA |
|  | Week4 | 4 (3.25, 4) | 4 (3, 4) |  |  | 2.11 (0.85, 3.83) | ** | 0.79 (-1.29, 2.68) | 0.49 |
|  | Week8 | 4 (3.25, 4) | 4 (3, 4) |  |  | 2.21 (0.93, 3.49) | *** | 0.15 (-1.54, 1.84) | 0.86 |
|  | Week12 | 4 (2.25, 4) | 3 (3, 4) |  |  | 1.62 (0.79, 2.45) | *** | 0.29 (-1.01, 1.60) | 0.66 |
| Stool frequency (times per week)^b^ | Week0 | 1 (1, 2) | 1 (1, 2) | 0.07 (-1.47, 1.60) | 0.93 | NA | NA | NA | NA |
|  | Week4 | 4 (2.25, 4.75) | 4 (3, 7) |  |  | 2.83 (1.59, 4.08) | *** | -0.40 (-1.76, 0.96) | 0.56 |
|  | Week8 | 4 (3, 7) | 4 (2, 6) |  |  | 2.56 (1.32, 3.80) | *** | 0.31 (-1.03, 1.64) | 0.65 |
|  | Week12 | 4.5 (3, 6.75) | 3 (2, 7) |  |  | 2.19, (0.99, 3.39) | *** | 0.74 (-0.64, 2.11) | 0.29 |
| MMSE^a^ | Week0 | 25.67 (4.05) | 25.27 (4.46) | 0.40 (-2.70, 3.50) | 0.80 | NA | NA | NA | NA |
|  | Week4 | 27.42 (2.50) | 27.27 (3.79) |  |  | 2.00 (0.95, 3.05) | *** | -0.25 (-2.08, 1.58) | 0.79 |
|  | Week8 | 26.33 (3.55) | 27.20 (4.00) |  |  | 2.93 (0.83, 3.03) | *** | -1.27 (-3.46, 0.93) | 0.26 |
|  | Week12 | 27.83 (2.59) | 26.73 (4.38) |  |  | 1.46 (0.31, 2.62) | * | 0.70 (-1.38, 2.78) | 0.51 |
| MOCA^a^ | Week0 | 19.08 (5.18) | 19.67 (5.43) | -0.58 (-4.45, 3.28) | 0.77 | NA | NA | NA | NA |
|  | Week4 | 20.83 (4.86) | 21.53 (5.00) |  |  | 1.87 (0.18, 3.55) | * | -0.12 (-2.95, 2.71) | 0.94 |
|  | Week8 | 22.83 (4.95) | 22.67 (4.91) |  |  | 3.00 (0.18, 3.55) | *** | 0.75 (-1.61, 3.11) | 0.53 |
|  | Week12 | 23.75 (4.35) | 22.93 (4.38) |  |  | 3.27 (1.99, 4.54) | *** | 1.40 (-1.11, 3.91) | 0.27 |
| PHQ-9^a^ | Week0 | 7.75 (4.14) | 6.93 (4.50) | 0.82 (-2.32, 3.96) | 0.61 | NA | NA | NA | NA |
|  | Week4 | 6.58 (3.40) | 6.27 (4.01） |  |  | -0.67 (-2.87, 1.53) | 0.55 | -0.50 (-3.21, 2.21) | 0.72 |
|  | Week8 | 8.00 (6.40) | 6.60 (4.00) |  |  | -0.33 (-2.73, 2.07) | 0.79 | 0.58 (-3.31, 4.48) | 0.77 |
|  | Week12 | 6.00 (5.78) | 6.67 (5.22) |  |  | -0.27 (-1.96, 1.43) | 0.76 | -1.48 (-5.14, 2.18) | 0.43 |
| GDS-15^a^ | Week0 | 5.92 (2.50) | 5.33 (2.82) | 0.58 (-1.35, 2.52) | 0.55 | NA | NA | NA | NA |
|  | Week4 | 5.75 (3.47) | 4.33 (3.11) |  |  |  | 0.17 | 0.83 (-1.13, 2.80) | 0.41 |
|  | Week8 | 4.42 (3.09) | 4.67 (3.42) |  |  | -0.67 (-2.02, 0.69) | 0.33 | -0.83 (-2.99, 1.32) | 0.45 |
|  | Week12 | 4.17 (3.01) | 4.40 (3.07) |  |  | -0.93 (-2.32, 0.46) | 0.19 | -0.82 (-3.08, 1.45) | 0.48 |
| GAD-7^a^ | Week0 | 4.08 (3.34) | 3.47 (3.20) | 0.62 (-1.78, 3.01) | 0.61 | NA | NA | NA | NA |
|  | Week4 | 3.25 (3.89) | 3.07 (2.76) |  |  | -0.40 (-1.84, 1.04) | 0.59 | -0.43 (-3.31, 2.44) | 0.77 |
|  | Week8 | 3.58 (4.83) | 3.67 (3.22) |  |  | 0.20 (-1.53, 1.93) | 0.82 | -0.70 (-3.89, 2.49) | 0.67 |
|  | Week12 | 3.17 (4.86) | 2.87 (2.75) |  |  | -0.60 (-2.18, 0.98) | 0.46 | -0.32 (-3.56, 2.93) | 0.85 |

^a^Data were presented as mean (SD), linear model was used in GEE analysis.

^b^Data were presented as median (IQR), ordinal logistic model was used in GEE analysis.

^c^Bristol score stands for the stool characteristics.

^d^Group effect represents the difference at baseline between two arms.

^e^Time effect represents change of measurement in the placebo arm compared with baseline.

^f^Group × time effect represents additional change of measurement in the FMT arm compared with placebo arm (additional change: the difference of measurement changes compared to baseline in two arms).

*: P < 0.05

**: P < 0.01

***: P < 0.001

**Supporting table 5. MENs topological attributes**

|  |  | **0W** | **4W** | **12W** |
| --- | --- | --- | --- | --- |
| Nodes | FMT.NR | 290 | 259 | 191 |
|  | FMT.R | 299 | 477 | 284 |
| Edges | FMT.NR | 1770 | 1748 | 603 |
|  | FMT.R | 2200 | 17284 | 2385 |
